# Effects of flow regimes on the interaction between granular flow and flexible barrier

**DOI:** 10.1371/journal.pone.0285559

**Published:** 2023-08-30

**Authors:** Siyou Xiao, Xingqian Xu, Haijun Wang, Dianxin Li, Zhongju Wei, Tengyuan Zhang

**Affiliations:** 1 School of Civil Engineering and Urban Planing, Liupanshui Normal University, Liupanshui, 553004, China; 2 Research Central for The Rural Security and Human Settlements of Guizhou, Liupanshui, China; 3 College of Water Conservancy, Yunnan Agricultural University, Kunming, 650201, China; National Sun Yat-sen University, TAIWAN

## Abstract

Flexible barriers are widely used to mitigate granular flows. In practice, flow regimes may keep changing along a flow path after the initiation of granular flows. The effects of flow regimes should be considered in the design of flexible barriers to intercept granular flow. In this study, flow regimes are divided into three types: dilute flow; dense flow; and quasistatic flow. The impact mechanisms of dense granular flows and dilute granular flows against flexible barriers are investigated using flume tests and the discrete element method. Influences of the ratio of the average particle size to the mesh size of a flexible barrier and particle segregation on the interaction between the flexible barrier and the granular flow are revealed. Differences of the impact mechanisms between rockfall and granular flow are compared. Results show that the impact force of dense granular flow against a flexible barrier will not increase linearly with the average particle size. The tensile force of the bottom cable is usually the maximum tensile force among all cables of the flexible barrier. Particle segregation will lead to increase in impact force of dense flows and tensile force of the upper cables. Impact force of the dilute granular flow increases with the average particle size. Different from the failure of a flexible barrier under the impact of the dense flow, the middle and upper cables are easier to break. Based on these findings, a useful reference for the future design of flexible barriers was proposed.

## 1 Introduction

Granular flow is a common geological hazard in mountainous areas. It increases disaster risks and safety maintenance costs for housing, transportation, and other infrastructure as a result of destruction of the geological environment caused by natural factors such as rainfall and earthquakes as well as human engineering activities such as housing construction, mining, and traffic facilities construction [1,2]. Flexible barriers have been widely used for mitigating small-scale granular flow because of the effective protection and better economic benefits.

Granular flow is composed of loose particulate matter. It exhibits the motion characteristics of a granular medium. The water content of granular flow could be high or even saturated. However, granular flow is not a fluid movement dominated by liquid phase but a fluidization movement dominated by the solid phase [3]. It has different collision and friction mechanisms at different shear rates and also has the characteristics of solid-liquid transformation [4]. Based on particle interaction, flow regimes can be divided into three types: quasistatic flow; dense flow; and dilute flow [5]. The interactions between the particles of the dilute flow are mainly collision instead of friction. The interactions between the particles of the dense flow are not just collision but also friction. The particles of the quasistatic flow are also close to static. The arching effect leads to blockage of the flow path. In general, the geological hazards disaster induced by the granular flow is the dilute flow or dense flow.

**Fig 1:**
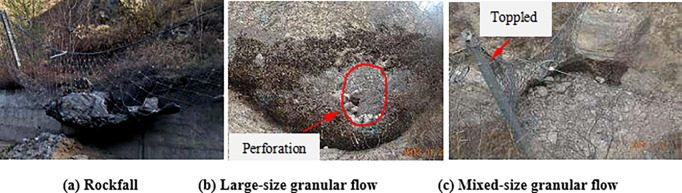
Different types of granular flow against flexible barriers.

**Fig 2:**
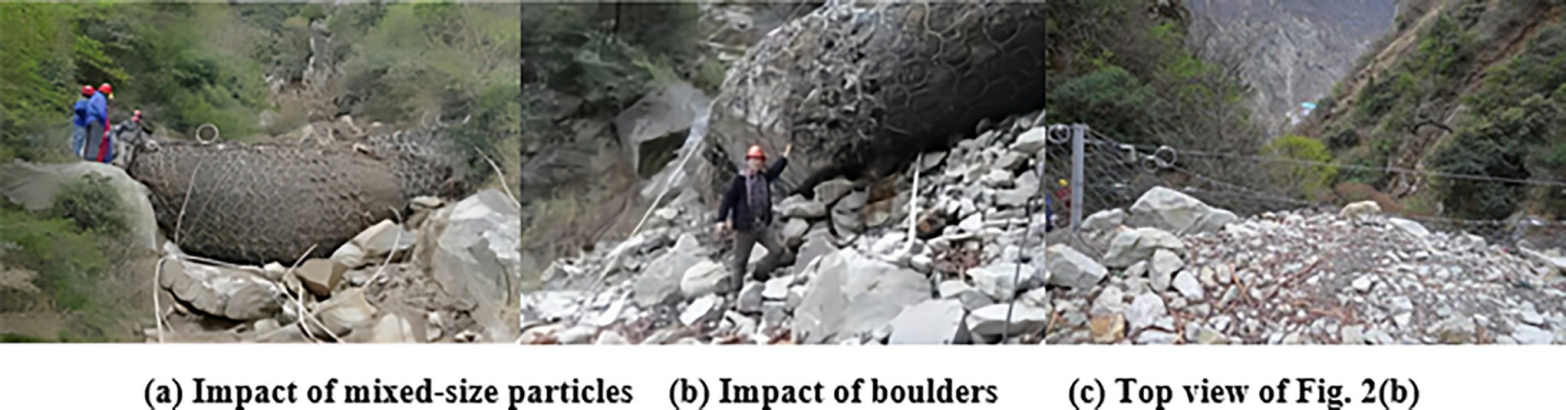
Granular flows with different particle size distributions against flexible barriers.

In practice, moreover, destruction of a flexible barrier may be caused by the impact of different flow regimes of the granular flow. There are two methods to estimate the protection capacity of the flexible barrier [3]: (1) The protection energy is a key design index of the structure of the flexible barrier; the protection energy is calculated using the sum of kinetic energy and potential energy of rockfalls or granular flows [6] [Fig 1(A) shows the deformation of the flexible barrier impacted by the rockfall]; (2) The hydrodynamic model is used for estimating the impact of the granular flow [7]. Fig 1(B) and 1(C) show the failure of flexible barriers impacted by the large-size granular flow and the mixed-size granular flow. Fig 1(B) shows that the mesh was broken because of the bullet effect of the large-size granular flow. The particles piled up from the bottom to the top of the flexible barrier, which led to the top cable falling off the column or column toppling. Fig 2 shows granular flows with different particle size distributions against flexible barriers. The deformation of mesh impacted by granular flow with boulders was obviously greater than the deformation caused by the mixed-size granular flow, as shown in Fig 2(A) and 2(B). The energy dissipators were close to failure because of the impact of granular flow with boulder, as shown in Fig 2(C). It illustrates that the flow states have a significant influence on the the interaction between granular flow and flexible barrier. In conclusion, the structure of the flexible barrier to intercept the granular flow should be designed according to the flow state of the granular flows.

At present, studies on the interaction between granular flow and flexible barriers appear less concerned about the changes in the granular flow regimes [8–13]. Notably, particle separation and flow regime change are barely taken account in the design of flexible barriers. Size distribution of granular flow is easy to obtain for initial parameters. Size distribution has an influence on the granular flow’s impact load [14]. Hence, investigating the influences of flow regimes on the interaction of granular flow and flexible barriers is critical for determining a flexible barrier’s optimal design. The granular flow regime is affected by particle-size distribution, flow distance, geomorphologic characterization, and so on. Its movement modes present a large discreteness. In this study, the granular flow is set as a dense flow with approximate continuous motion and a dilute flow with obvious discrete motion. By comparing the impact load of granular flow under the same initial mass conditions and initial potential energy conditions, the influences of the change in granular flow regimes on the impact load of the granular flow and the damage characteristics of the flexible barrier are revealed.

## 2 Experiment and simulation methodology

### 2.1 Flume test

**Fig 3:**
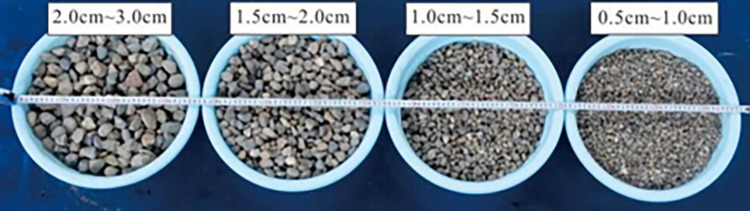
Composition of granular flow.

**Fig 4:**
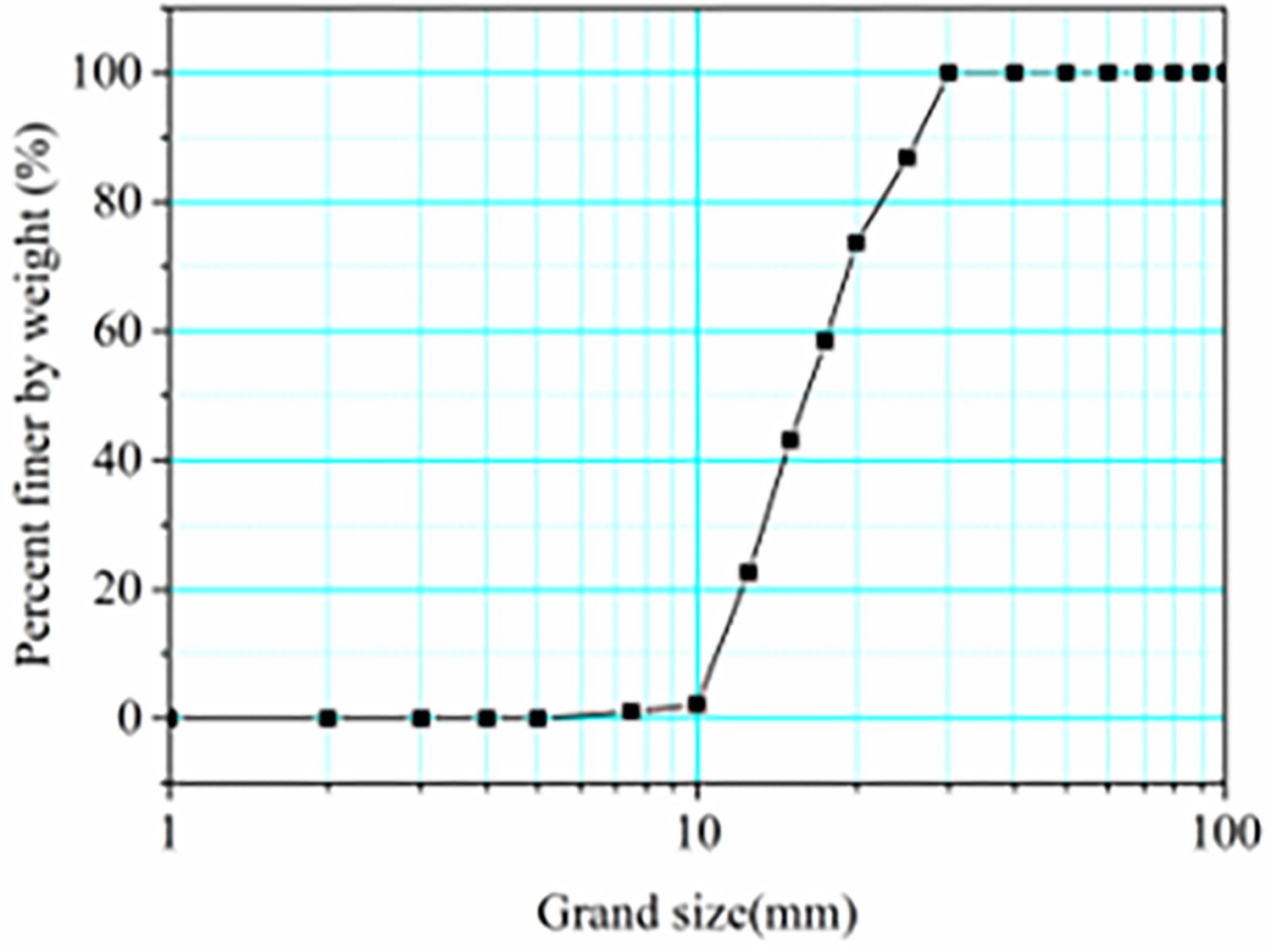
Grain size distribution of the test materials.

**Fig 5:**
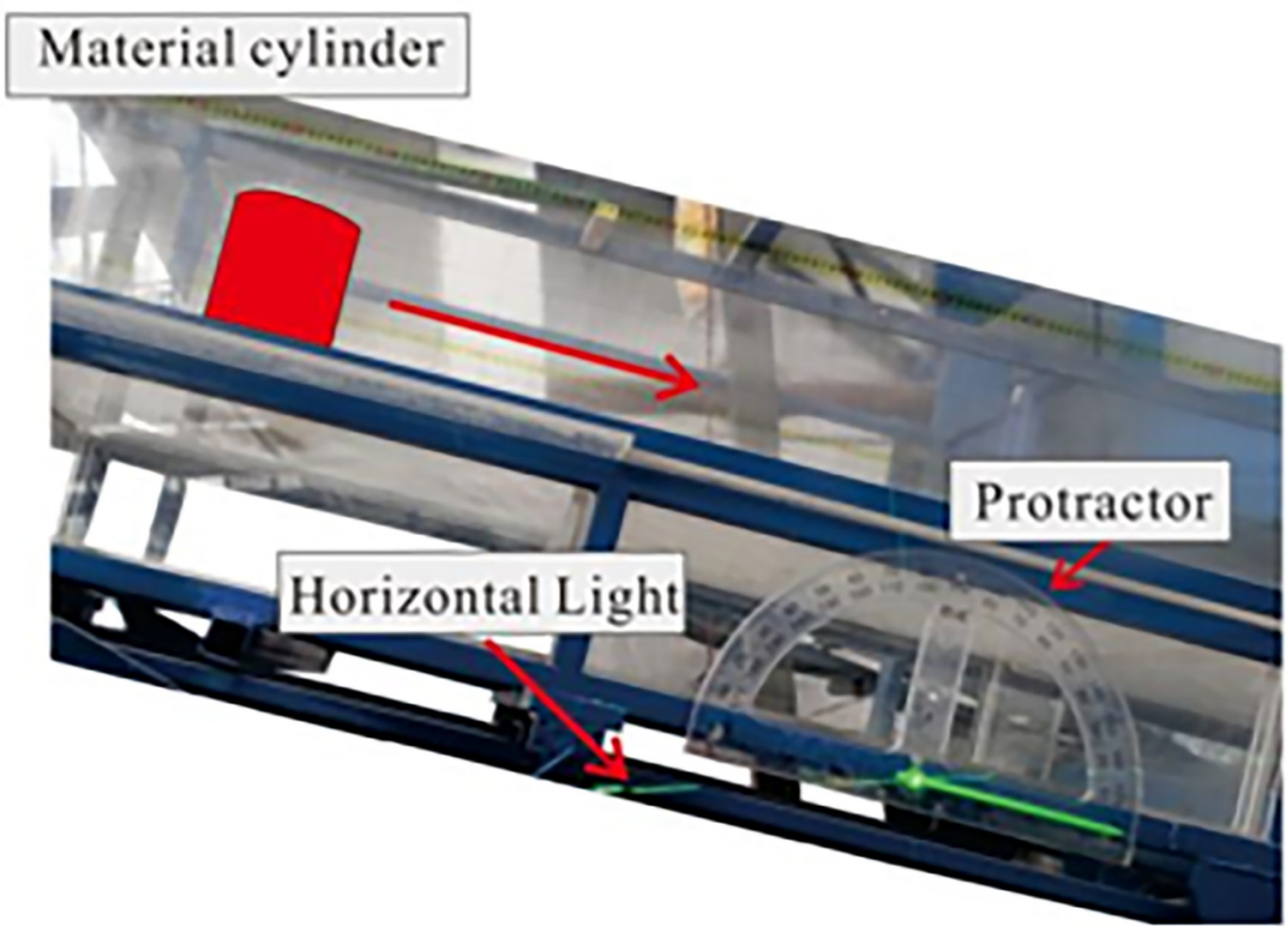
Setup of the inclined slope experiment.

**Fig 6:**
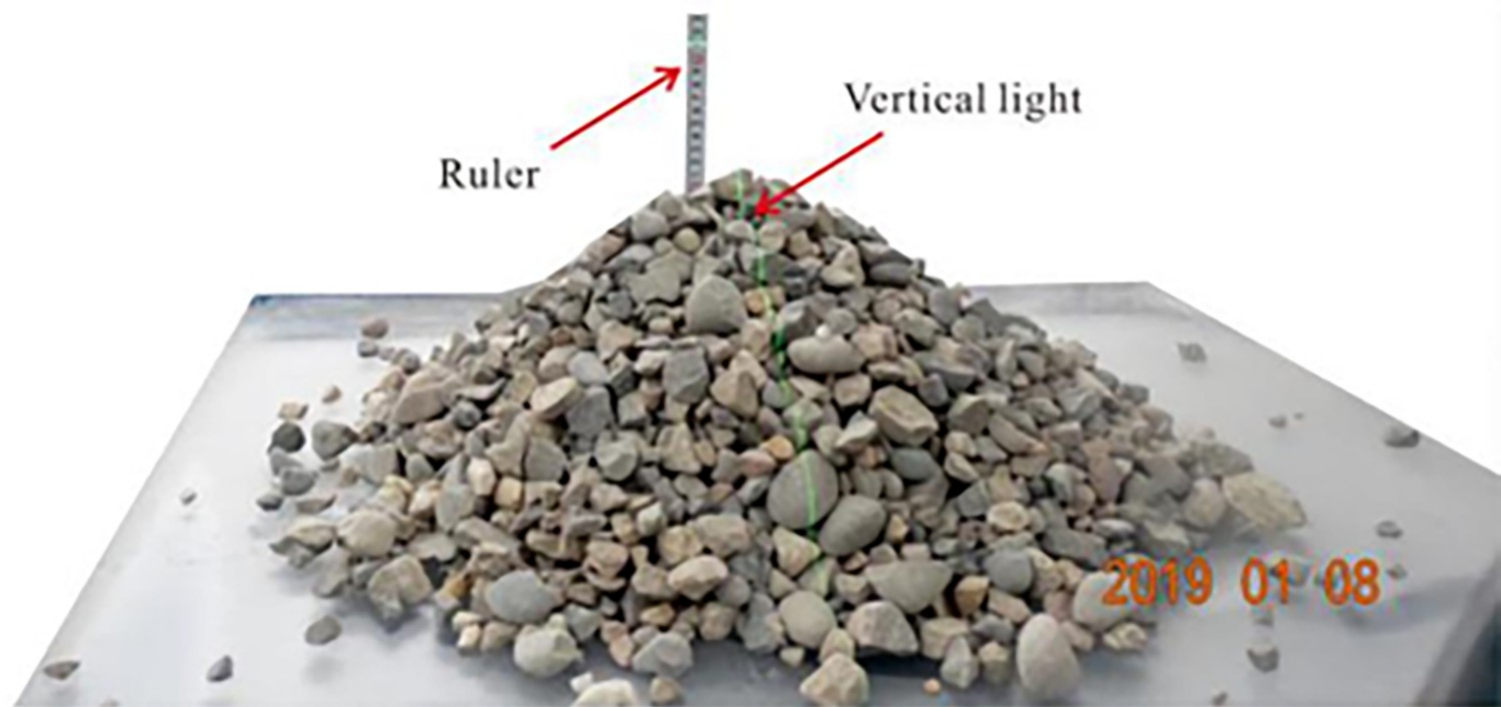
Accumulation experiment.

Particle size is mainly distributed from 5 to 50 cm in the field. In some cases, the granular flow may contain boulders with a radius of more than 50 cm. The granular flow material is designed according to a similarity ratio of about 1:10. The test materials are composed of natural gravel, as shown in Fig 3. In this study, the range of particle size is 0.5 cm~3 cm. Bulk density and grain density are 1636 and 2650 kg/m^3^. Volume of the granular flow is 0.045 m^3^. The curve of the particle size distribution is plotted in Fig 4. The average particle size (*D*_50_) is 1.52 cm. The friction parameter *δ* between the test materials and the base of the flume was obtained from the tiling experiment conducted by Xiao et al. (2020) [15]. The inclined slope experiment is shown in Fig 5. The angle of repose *φ* was tested using the accumulation experiment, as shown in Fig 6. Based on the tests, the friction parameter *δ*_1_ and angle of repose *φ* is 21° and 40.5°, respectively.

**Fig 7:**
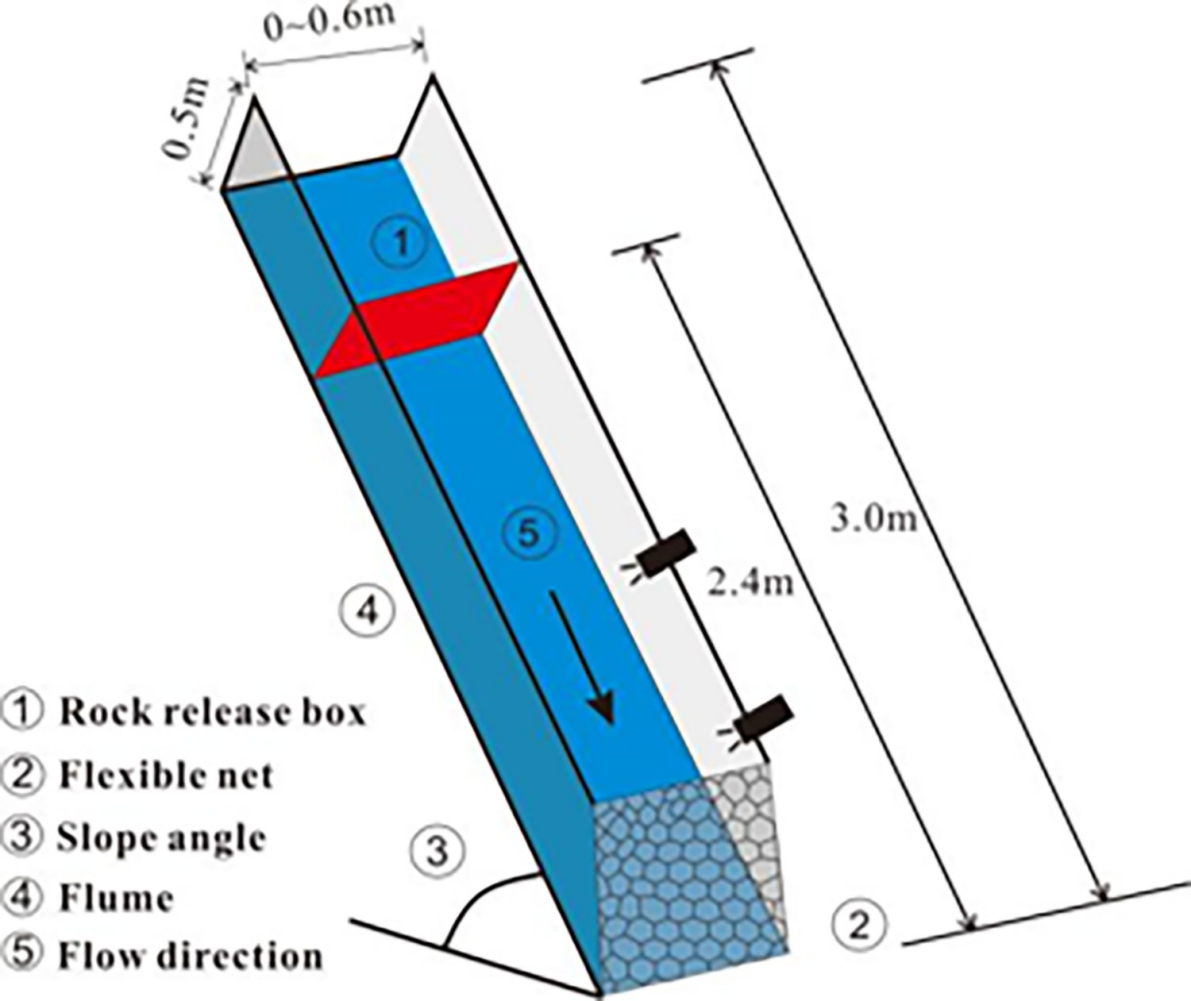
Diagram of the flume.

**Fig 8:**
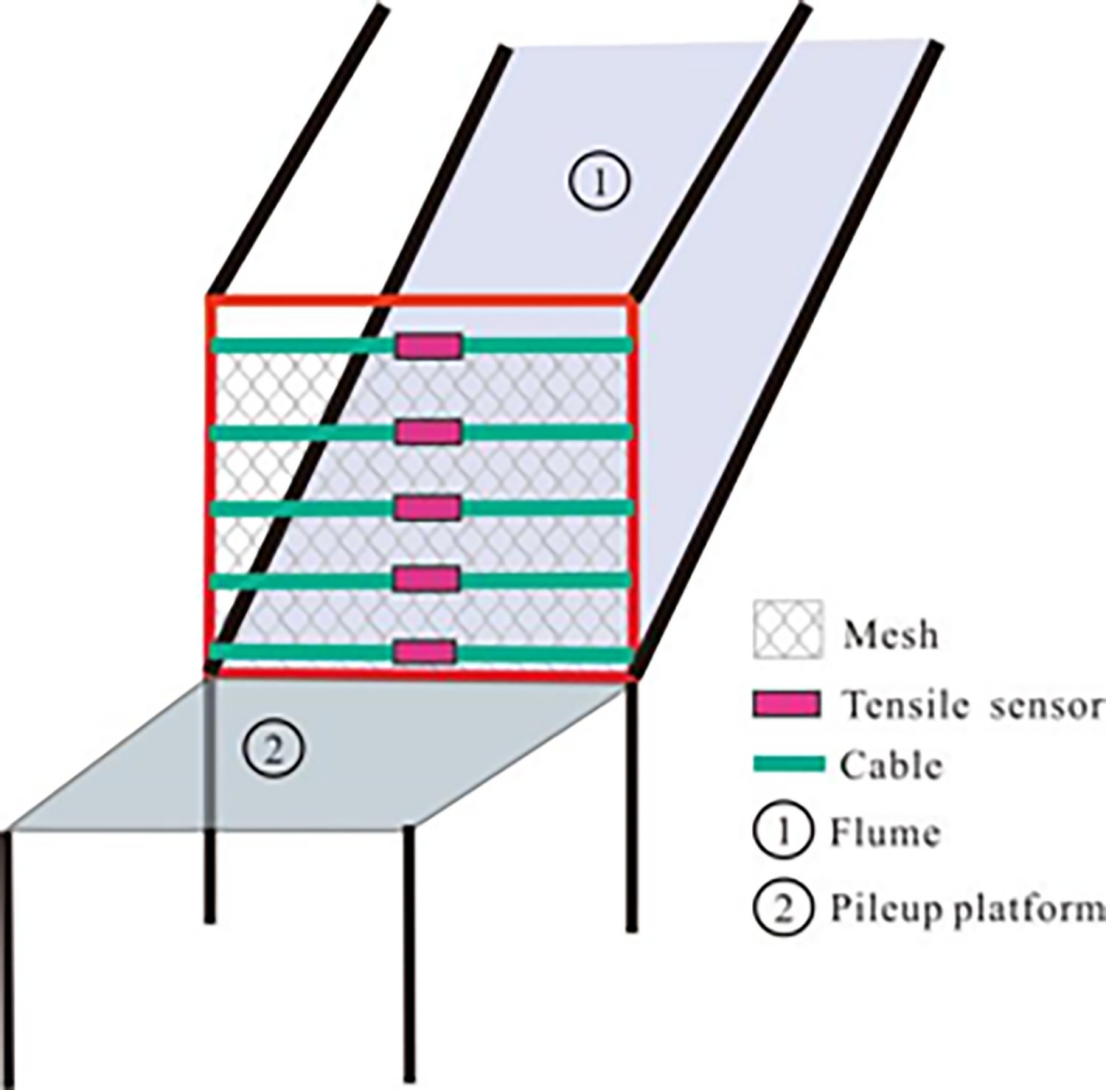
Layout of the flexible barrier.

The diagram of the flume is shown in Fig 7. The length and width of the flume is 2.4 and 0.6 m, respectively. The height of the flume is 0.5 m. In order to reduce the scale effect, small-scale flume tests to restore field conditions are often constrained by the principles of geometric similarity, material similarity, dynamic similarity, etc. According to the geometric similarity theory, as the similarity ratio of 1:10 is adopted, the corresponding path length of the granular flow is 24 m. The corresponding width and corresponding volume are 6 and 45 m^3^, respectively. The corresponding field granular flow is a small slope granular flow or a V-shaped gully granular flow. The elastic modulus of the cables is 39.92 GPa. The tensile strength is 855.1 N/mm^2^. The cables were numbered Cab 1~Cab 5 from the bottom to top. The spacing between each cable is 10 cm. The elastic modulus of the mesh is 38.5 GPa. The mesh was bound on the cable to form an interception net. The tensile sensors are connected with each cable to monitor the tensile force of each cable, as shown in Fig 8. The dynamic similarity is usually evaluated by the Froude number and Savage number. Froude number *Fr* can be written as follows:

$$Fr=\frac{v}{\sqrt{gh\cos\theta }},$$ (1)

where *v* is the flow velocity, *h* is the flow height, and *θ* is the slope angle,°. Savage number *Ns* can be expressed as

$$N_{S}=\frac{\rho_{m}{\dot{\gamma }}^{2}\delta^{2}}{(\rho_{m}-\rho_{f})gh\tan\varphi \cos\theta },$$ (2)

where *γ* is the shear rate, 1/s, and *φ* is the angle of repose,°. *δ* is the average particle diameter. *ρ*_m_ is the density of granular flow. *ρ*_f_ is the density of flow.

### 2.2 Numerical simulation

**Fig 9:**
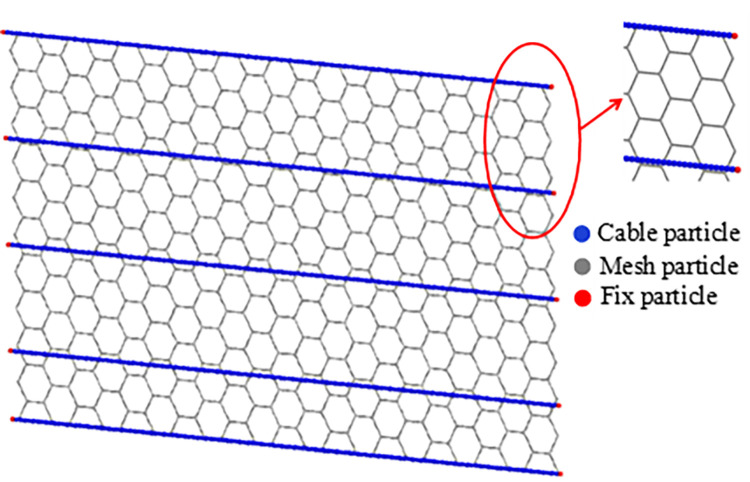
Numerical model of flexible barrier.

The discrete element method was used for simulating the interaction between the granular flow and the flexible barrier. The contact model of the flexible barrier and the granular flow are, respectively, Hertz–Mindlin with bonding model and Hertz-Mindlin (no-slip) [16]. Parameters for the numerical simulation were calibrated by accumulation and flume physical tests of the granular flow impacting the flexible barrier; the particle rolling friction coefficient of the granular flow is 0.16, the restitution coefficient is 0.5, the restitution coefficient between the granular flow and the walls of the flume is 0.5, and the rolling friction coefficient is 0.05. Here, the density of the wall of the flume is 1500 kg/m from the material library of EDEM, the Poisson’s ratio is 0.4, the shear modulus is 100 MPa, the shear modulus of the test materials is 850 MPa, and the Poisson’s ratio is 0.2. The numerical average radius of granular particles is the same as the particles’ radius of the physical test materials. The numerical model for the flexible barrier consists of three types of particles: fixed particles, mesh particles, and cable particles, as illustrated in Fig 9. Fixed particles are used to secure the ends of the cables and remain in a fixed position to keep the barrier in place. To reduce the number of particles, the numerical average radius of cable and mesh particles is slightly larger than the physical radius of the cables and meshes. The radius of fixed particles is equal to the radius of cable particles.

**Fig 10:**
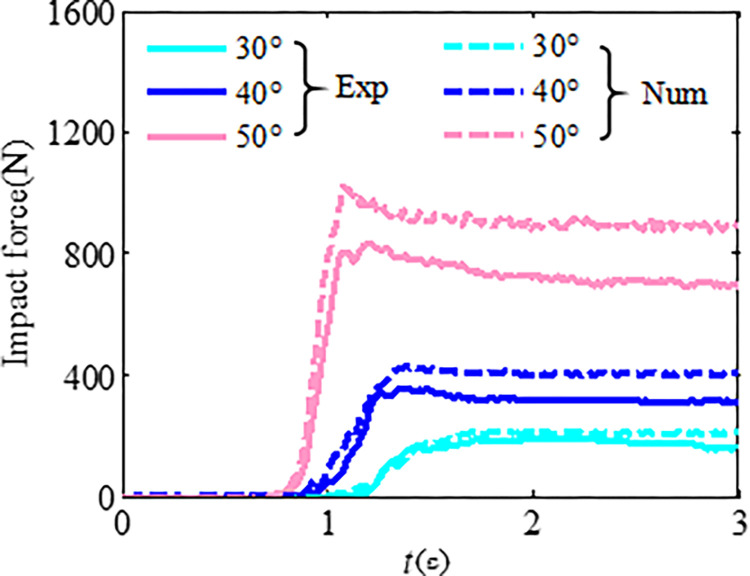
Comparison between the physical experimental and numerical results.

The discrete element bonding parameters of the cables and mesh with initial deformation need to be reduced on the basis of the elastic stiffness from the physical test; then, the optimal discrete element simulation parameters can be obtained by comparing the numerical calculation and experimental calculation results using the numerical calibration test. In the case of 30°, 40°, and 50° slopes, the load of the granular flow impacting the flexible barrier from the numerical simulation was compared with the physical experimental results, as shown in Fig 10. The numerical simulation and physical test results are consistent when the slope is less than 40°. In contrast, the discrete element calculation results are greater than the physical experimental results. The main reason may be that the soil arching effect of granular flow particles could not be effectively simulated using the ideal sphere particles.

**Fig 11:**
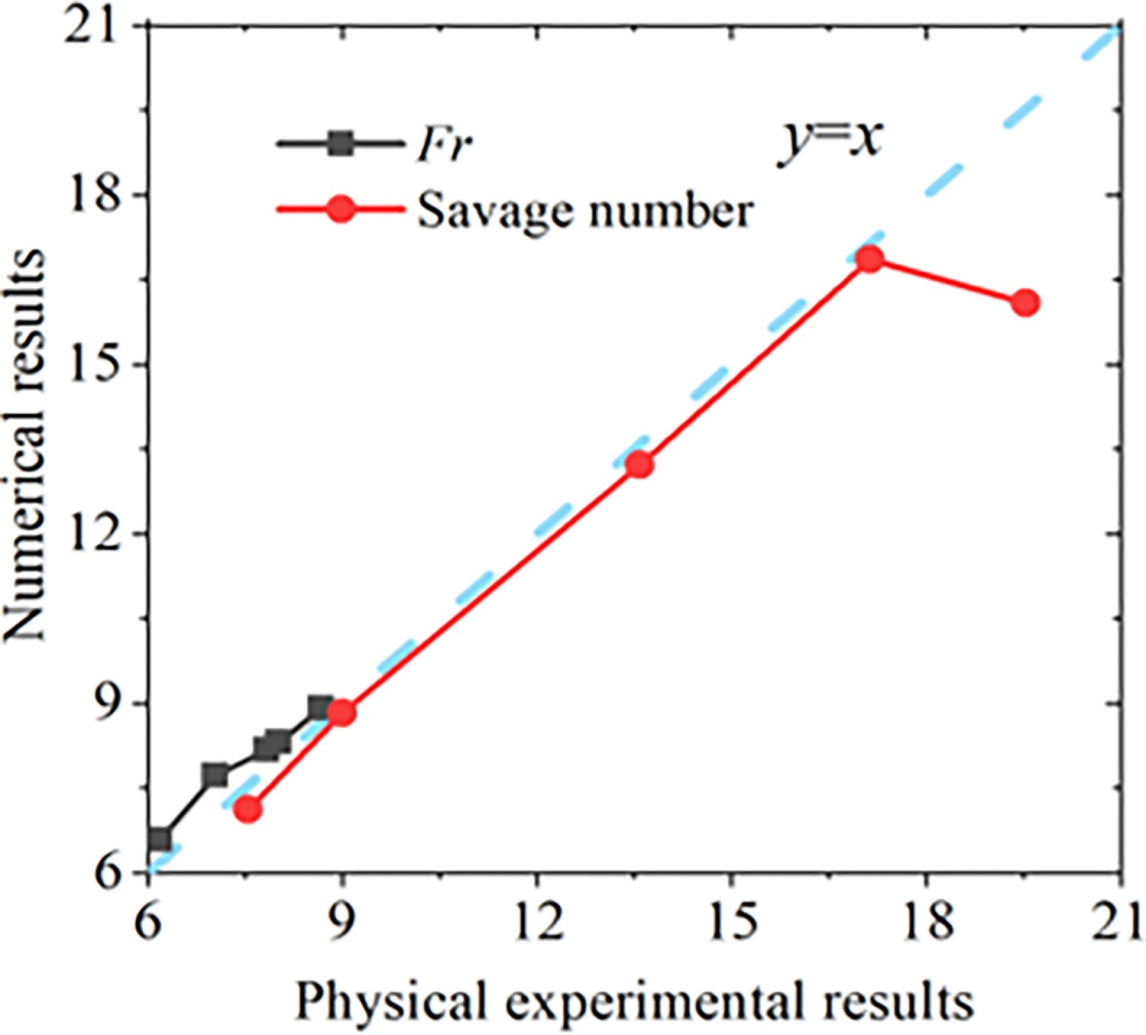
Comparison of Froude and Savage numbers between the physical experimental and numerical results.

In this study, if pileup height ceased to increase, the flow velocity *v* and flow height *h* were measured at the moment (time) when the maximum pileup height has just been reached. Otherwise, *v* and *h* are the average velocity and average height of the maximum velocity gradient. The key indexes of dynamic similarity of the numerical tests and physical tests are listed in Table 1. The comparison of *Fr* number and Savage number between the physical experimental and numerical results is presented in Fig 11.

**Table 1:** Comparison between Froude and Savage numbers.

Slope angle/°	Flow height/m		Flow velocity/m·s^-1^		Fr number		Savage number	
	Numerical tests	Physical tests	Numerical tests	Physical tests	Numerical tests	Physical tests	Numerical tests	Physical tests
30	0.027	0.023	3.15	2.73	6.58	6.18	16.08	19.54
35	0.031	0.028	3.86	3.34	7.73	7.04	16.86	17.13
40	0.037	0.035	4.31	4.02	8.18	7.84	13.22	13.59
45	0.046	0.044	4.69	4.43	8.31	8.02	8.82	9.00
50	0.055	0.052	5.25	4.97	8.91	8.68	7.12	7.55

According to the flume tests conducted by Zhou and Song [17–19], the ranges of *Fr* number and Savage number is 3 to 10 and 0.001 to 12. In this study, the range of *Fr* number is less than 10. The Savage number is less than 20. Hence, it is in a reasonable range to restore field conditions. The errors of *Fr* and Savage numbers between the physical tests and numerical tests is less than 20%, which illustrates that the flow process of granular flow can be simulated effectively using the discrete element method. In general, the physical test results of the impact load and the flow characteristics could be simulated using the discrete element method. The calibrated parameters of discrete element model material and flexible barrier discrete element contact model are shown in Tables 2 and 3.

**Table 2:** Material parameters of granular flow and flexible barrier.

Material parameters	Density (kg/m^3^)	Poisson’s Ratio	Shear Modulus (Pa)	Particle Radius/cm
Granular particles	2650	0.2	8e^8^	0.76
Cable particles	7900	0.15	1e^9^	0.25
Mesh particles	7900	0.15	1e^9^	0.1
Fixed particles	1e15	0.15	1e^9^	0.25
Chute	2500	0.2	1e^8^	/

**Table 3:** Bonding parameters of the flexible barrier.

Contact Particles	Per Square Normal Stiffness (N/m^3^)	Per Plane Tangential Stiffness (N/m^3^)	Critical Normal Stress (Pa)	Critical Tangential Stress (Pa)
Support rope/support rope	4e10	1e10	3e12	1e12
Support rope/mesh	2e10	1e10	3e12	1e12
Mesh/mesh	2e10	1e10	3e12	1e12
Support rope/fixed particles	4e10	1e10	3e12	1e12

### 2.3 Test program

To investigate the effects of the changes in the flow regimes on the interaction between granular flows and flexible barriers, wide-grade gravel and uniformly graded gravel were employed. The average particle radius is varied from 0.5~18.9 cm to cover the flow regimes, including dense flow, dilute flow, and rockfall. The distribution of particle size may have an influence on the passing rate of the particles through a flexible barrier. Hence, different ratios of mesh size to particle size are used to reveal the effects of the passing rate of the particles through a flexible barrier. The mesh diameters of the flexible barrier is 1, 3, and 5 cm, respectively. The slope angle of the flume *θ* is changed from 30° to 50°. Using the physical and numerical flume tests, the influences of average particle size of dense flow and particle separation on the tensile force of flexible meshes were released. The differences of the tensile for flexible barrier among the impact of dense flows, dilute flows, and rockfalls were revealed by the numerical flume tests.

## 3 Influence of dense flow state on the interaction between flexible barrier and granular flow

There is still no unified standard for the quantitative distinction between dense flows and dilute flows; it is generally believed that the transition of granular flow from dense flow to dilute flow is affected by particle size, opening width, initial velocity, and flow [20,21]. The flow state of granular flow is dilute flow when the Froude number is greater than 10, and the contact force between the particles is dominated by collision force [5]. The ratio of the particle size to the flow depth must be greater than 1. If the ratio of the particle size to the flow depth is less than 2, it means there may not be two particles in the direction of flow height. In this case, the interaction between particles is mainly collision. Hence, the flow regime is close to dilute flow. Based on the above studies, the main criteria for judging dilute flow and dense flow are as follows: (1) the Froude number *Fr* is greater than 10; (2) the ratio of the particle size to the flow depth is less than 2.

### 3.1 Influences of particle size

Particle size distribution has an influence on the impact load of the granular flow. Gao et al. and Wu et al. pointed out that particle shape and volume jointly affected the impact load of dry granular flow on retaining walls [22,23]. Cui et al. investigated the impact load of granular flow against a rigid retaining wall with a ratio of particle size to flow depth from 0.15 to 0.62. It was found that the pulse load of the granular flow increases with the particle size [24]. Song et al. carried out impact tests of granular flow against flexible barriers (particle sizes ranging from 0.6 to 87 mm) using a geotechnical centrifuge. It was revealed that particle size has little influence on impact load of granular flow against a flexible barrier, which is different from the interaction between the granular flow and the rigid retaining wall [14].

The bulk density and angle of repose of four groups’ granular particle materials with different particle sizes, as shown in Fig 3, were measured. The corresponding particle size ranges of granular particle material were numbered *R*_1_~*R*_4_. The particle size distribution of the granular flow used in the granular flow impact test was numbered as *R*_m_. The bulk density and internal friction angle are listed in Table 4. It can be seen that the bulk density of the granular flow increases in particle size. It indicates that the impact load of the granular flow is also different for the same initial volume. The internal friction angle corresponding to the different particle size ranges of granular flow also were varied with particle size, but the test results did not show an obvious increase in the particle size range. Hence, the influence of particle size distribution on the initial potential energy and stacking friction coefficient of debris flow is relatively small.

**Table 4:** Densities and internal friction angles of test materials with different size.

Group	Particle Size Range (cm)	Bulk Density (kg/m^3^)	Internal Friction Angle (°)
*R*_1_	0.5~1.0	1542	38.6
*R*_2_	1.0~1.5	1608	39.1
*R*_3_	1.5~2.0	1670	41.4
*R*_4_	2.0~3.0	1767	40.8
*R*_m_	0.5~0.3	1636	40.5

**Fig 12:**
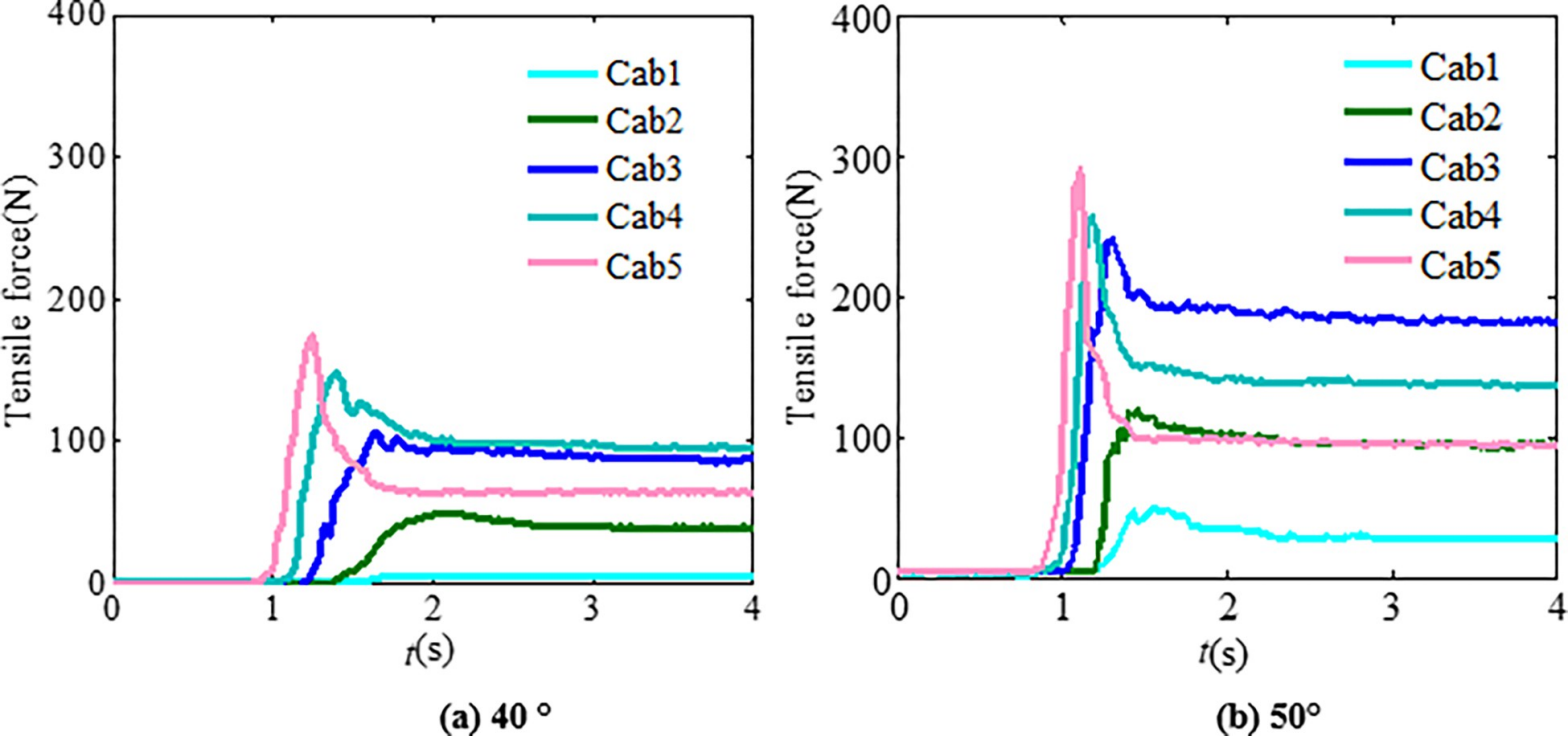
Support rope tension time-history curves (particle size range is 1 cm~2 cm).

**Fig 13:**
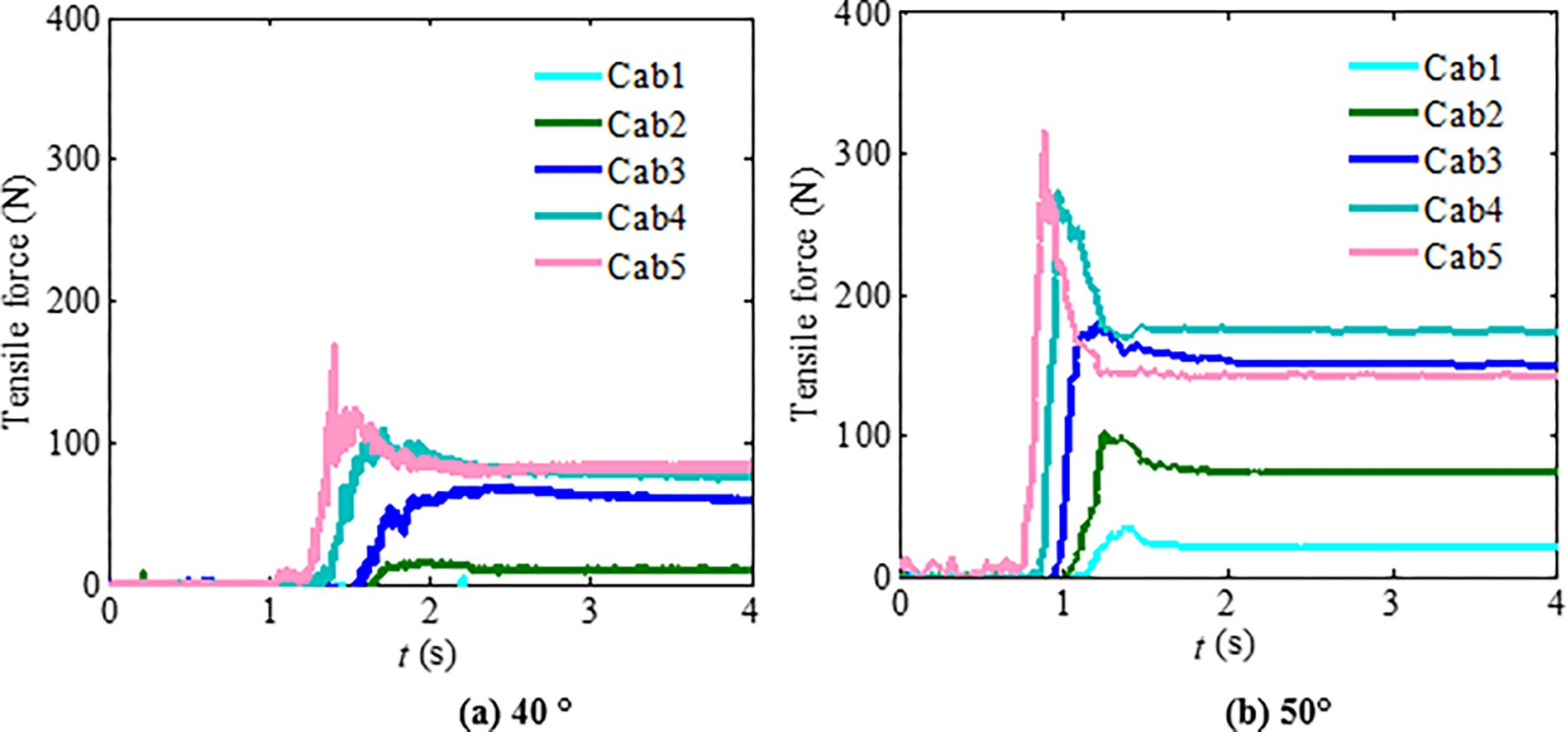
Support rope tension time interval curves (particle size range is 2 cm~3 cm).

According to the physical test results, the tension force time history of granular flow impacting flexible barriers with a particle size range of 1~2 cm and 2 ~ 3 cm is as shown in Figs 12 and 13. By comparing the time interval curves of the support rope under the impact of granular flow with a particle size range of 0.5~3 cm, it is shown that there are only a few changes in the force of the cables under the impact of granular flow (less than 10%). Hence, it is not necessary to design a flexible barrier, especially when the average particle size increases within two times if the particle size is uniform [16].

According to mechanics of cable structures, the impact load *F*_*i*_ against Cab *i* can be written as [15]:

$$F_{i}=2T_{i}\frac{4f_{i}}{\sqrt{l_{i}^{2}+{\left(4f_{i}\right)}_{}^{2}}}$$ (3)

where, *f*_*i*_ is the deflection of Cab *i*, *T*_*i*_ is the tensile force of Cab *i*, *l*_*i*_ is the length of Cab *i*.

Jiang et al. (2020) monitored the deflection using the images of the impact process [13]. However, it is hard to monitor the deflection of middle point because the pileup particle covered the bottom cable. Moreover, the measurement accuracy is also affected by factors such as camera placement installation position and installation angle. Song used a laser rangefinder to measure the deflection, but the displacement of cable was assumed to be the displacement released by spring [14]. Based on these monitoring methods, a method is proposed to estimate deflection based on the relationship between the final deflections *f* and the residual tensile force *T*_residual_. The physical test results of final deflection and the residual tensile force are listed in Table 5.

**Table 5:** The final deflection and residual tensile force.

Cable ID	30°		35°		40°		45°		50°	
	*f*/cm	*T*_residual_/N	*f*/cm	*T*_residual_/N	*f*/cm	*T*_residual_/N	*f*/cm	*T*_residual_/N	*f*/cm	*T*_residual_/N
Cab 1	0	0	0	0	0	0	1.2	16.0	3.2	28.6
Cab 2	0	0	0	0	3.0	23.4	4.9	41.2	4.9	91.9
Cab 3	3.2	32.0	4.3	53.5	4.6	68.4	7.8	111.5	7.9	178.6
Cab 4	4.7	69.8	4.7	77.4	4.9	76.1	7.2	90.9	8.3	133.1
Cab 5	4.0	49.2	4.2	44.3	4.6	50.2	6.0	46.8	6.4	93.5

Fit the final deflection and residual tension, and the corresponding fitting formula is:

$$f=5.07\mathrm{atan}(0.03T)+1.18;,\;R^{2}=0.92$$ (4)

**Fig 14:**
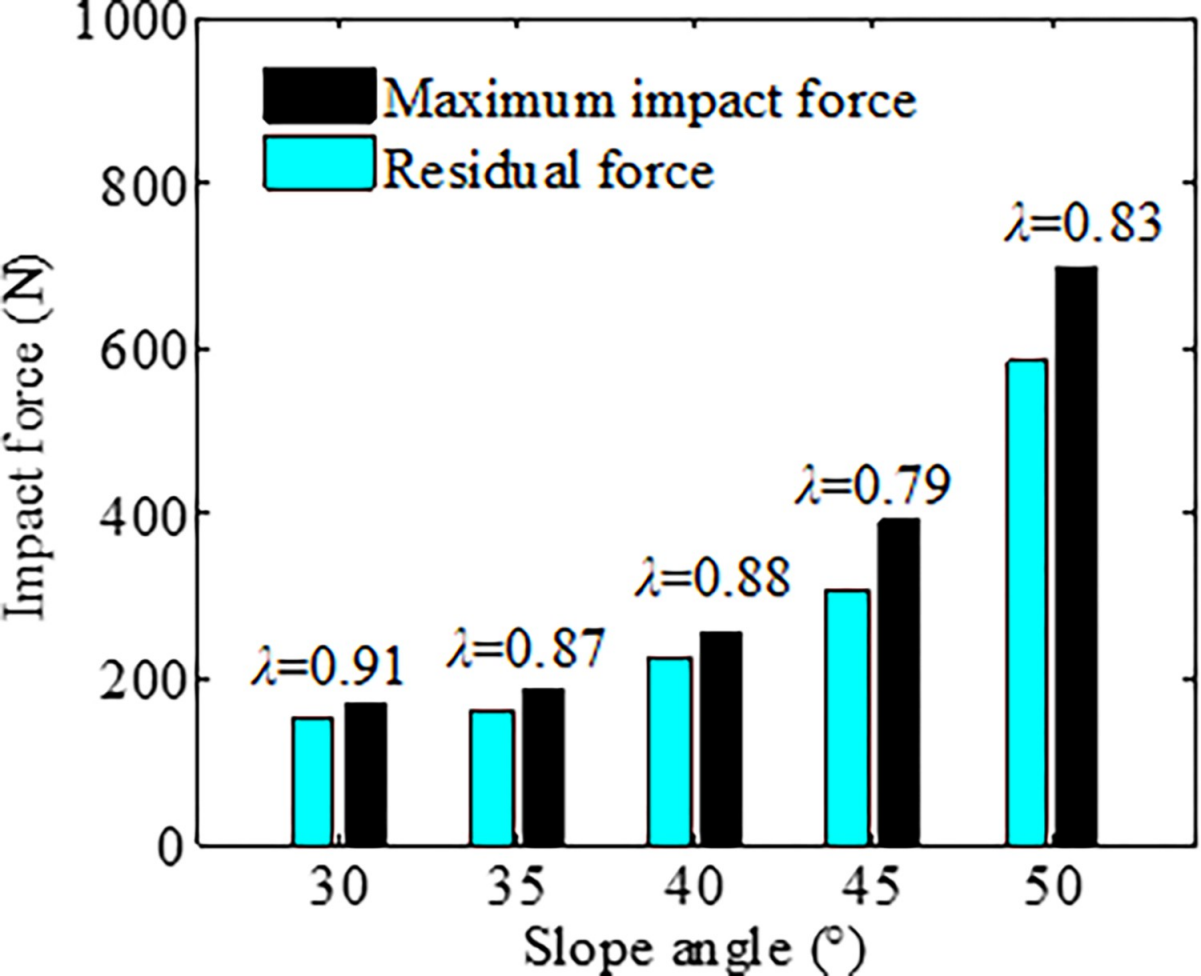
The relationship between impact force *F*_max_ and the residual force *F*_residual_.

Substituting Eq 4 to Eq 3 yields the impact load *F*_*i*_. The maximum impact force *F*_max_ and the residual force are listed in Table 6. The ratio λ of maximum impact force *F*_max_ to the residual force is plotted in Fig 14. It indicated that λ responding to a smaller slope angle is greater than that responding to a bigger slope angle. The maximum λ is 0.79. It illustrates that the the maximum impact force can be estimated using the resident force.

**Table 6:** The maximum impact force *F*_max_ and the residual force.

Slope angle/°	Maximum impact force *F*_max_/N	Residual force/N
30	167.9	152.9
35	183.8	160.8
40	256.1	225.7
45	390.5	308.2
50	697.2	582.7

**Fig 15:**
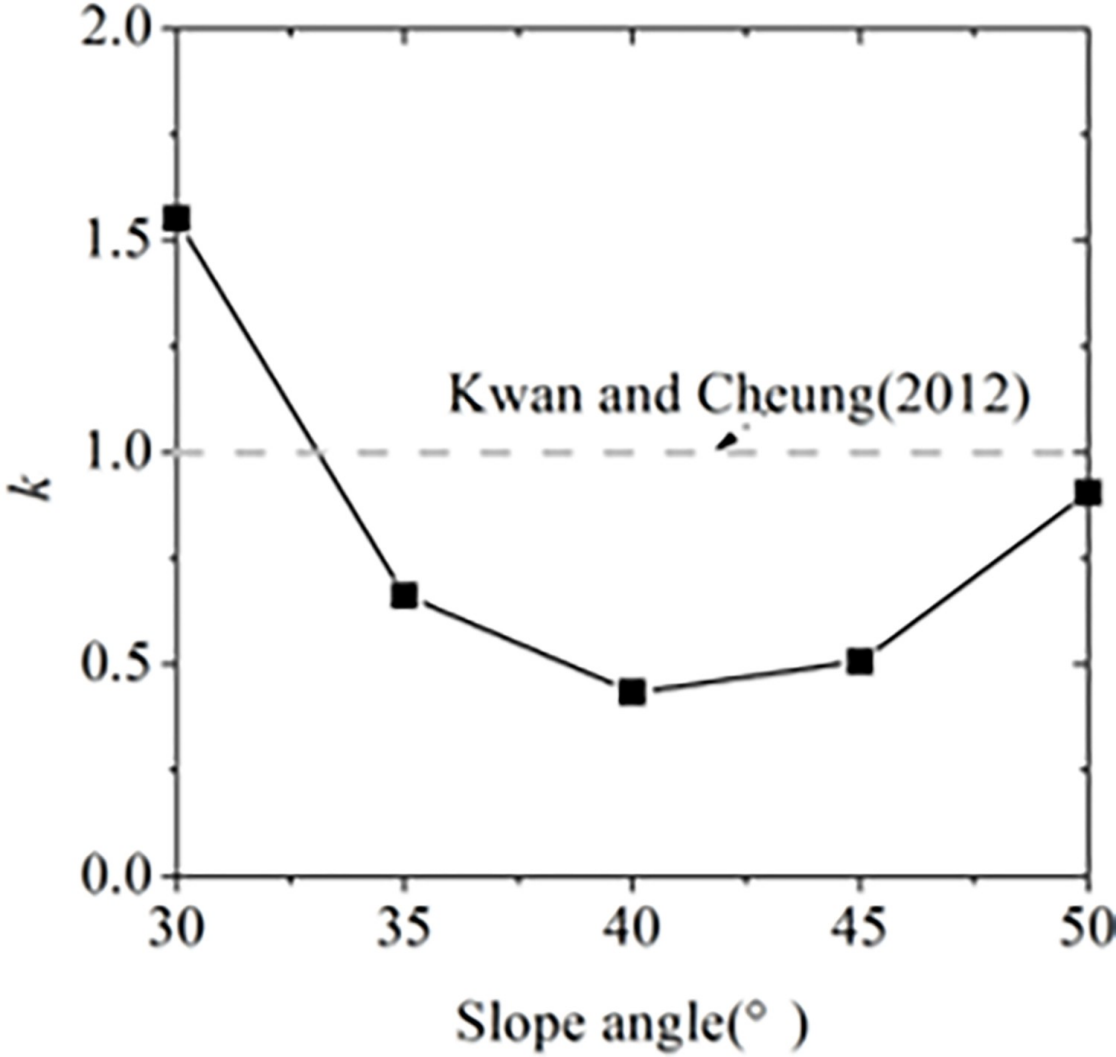
Empirical static-hydro coefficient.

**Fig 16:**
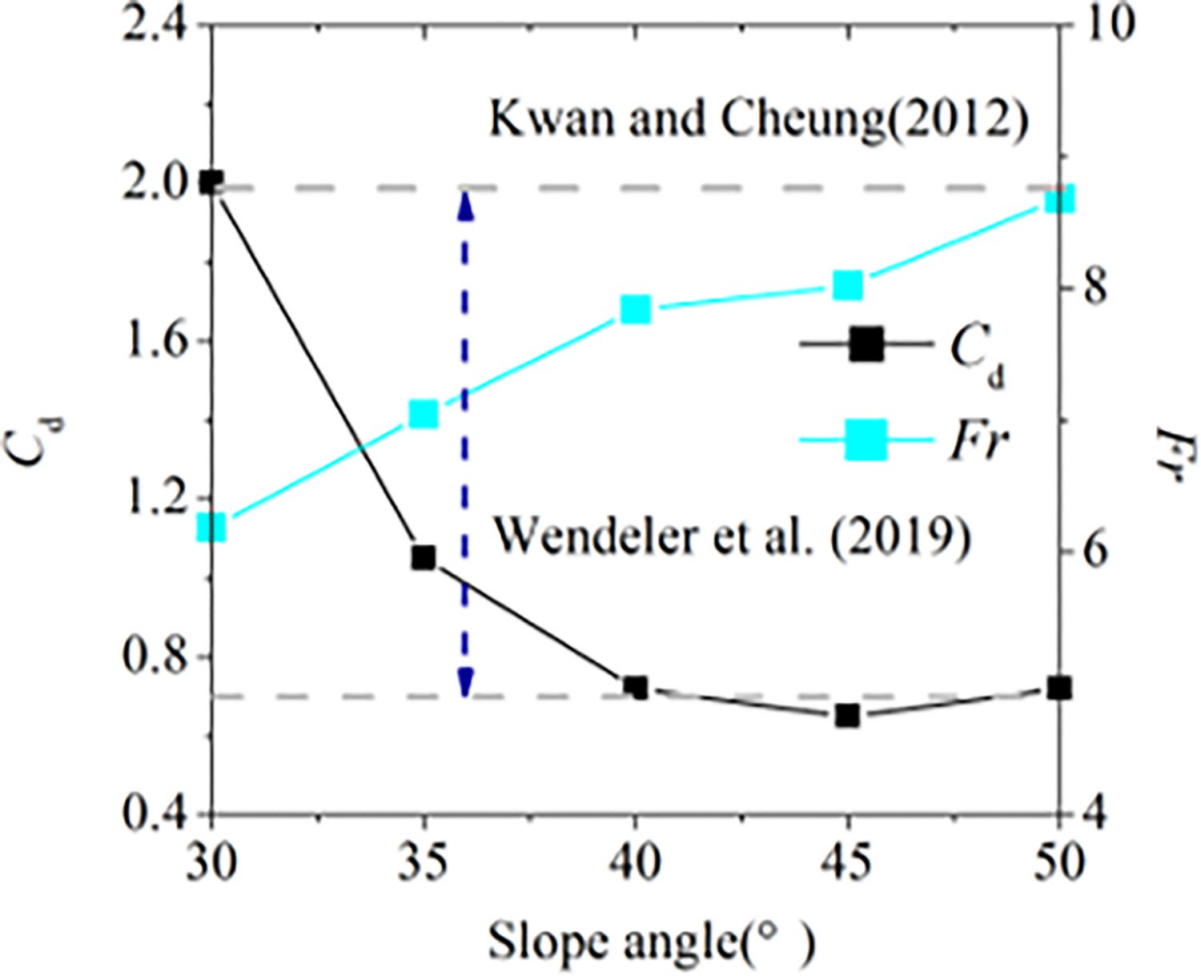
Dynamic coefficient and Froude number.

**Fig 17:**
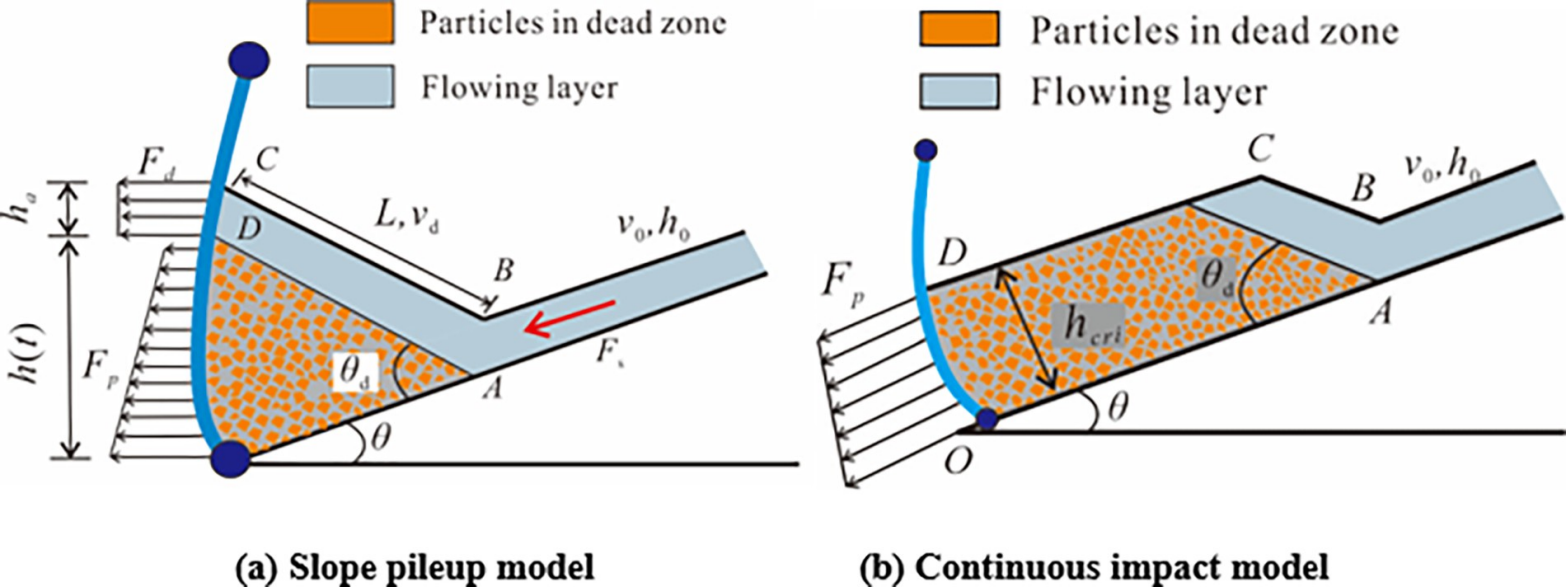
Impact models of dense flow against flexible barriers.

**Fig 18:**
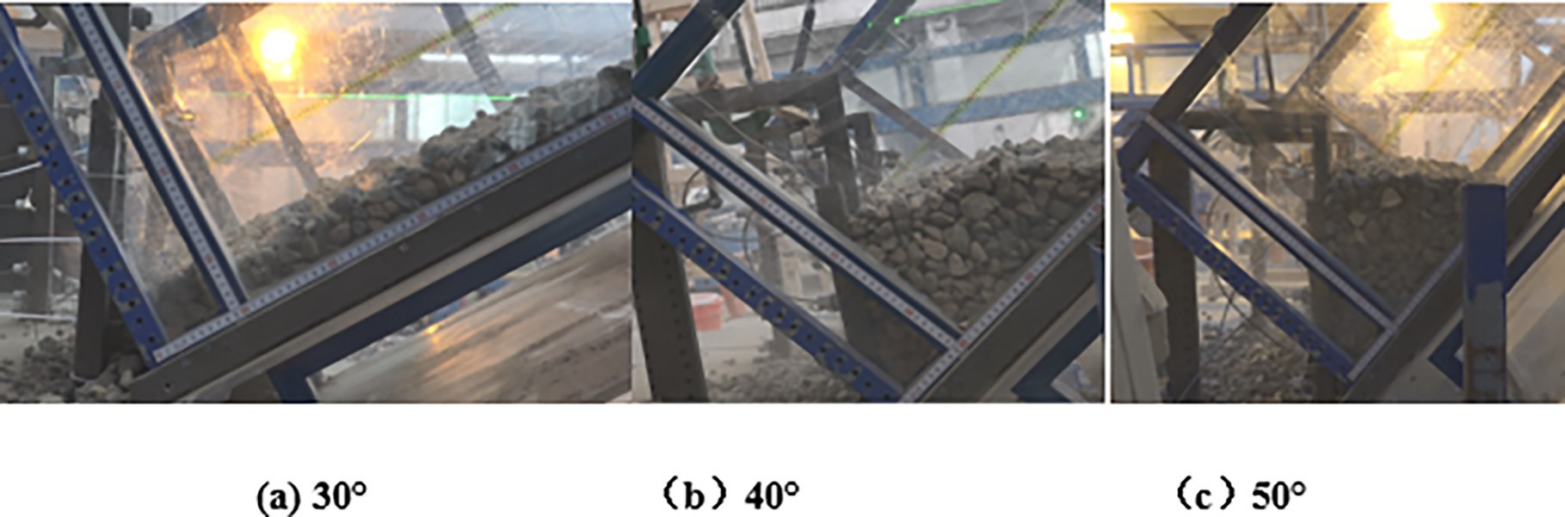
The pileup types corresponding to different slope angle.

The maximum impact load *F*_m_ of dense flow can be estimated by the hydrostatic model or hydrodynamic model as follows:

$$F_{\max}=\frac{1}{2}k\rho gh_{{}_{\mathrm{deposit}}}^{2}w\sin\theta_{1},$$ (5)

$$F_{\max}=C_{d}\rho v_{}^{2}h_{}w\sin(\theta +\theta_{1}),$$ (6)

where *k* is the empirical static coefficient, *h*_deposit_ is the final pileup height, *w* is the width of is the width of the barrier, *θ*_1_ is installation angle of flexible barrier, and *C*_d_ is the empirical dynamic coefficient. Figs 15 and 16 show the ranges of the empirical static coefficient *k* and the empirical dynamic coefficient *C*_d_ based on the physical tests of the impact of granular flow against tiled flexible barrier which is perpendicular to the slope surface and vertical flexible barrier which is perpendicular to the earth’s surface. The indexes of the impact process and pileup are listed in Table 7. The empirical static-hydro coefficient is 0.5 to 2.5. The dynamic coefficient is 0.5 to 1.5 when the Froude number is 4 to 10. It is consistent with the value range of 0.7~2.5 proposed by Wenderler et al. [10] and Kwan et al. [25] basically. According to the pileup characteristics, the impact models of dense flow against flexible barriers can be divided to two models: slope pileup model and continuous impact model as shown in Fig 17. If the flowing layer can impact flexible barrier continuously at the end of the impact process, it is called as continuous impact model. The impact velocity *v*_d_ and the flow height *h*_a_ on the top of dead zone is greater than 0. The pileup up height *h*(t) and the length of the top of dead zone *L* keep increasing. The impact force *F* of granular flow against flexible barrier consists of three parts:

$$F=F_{s}+F_{d}+F_{p},$$ (7)

where, *F*_s_ is the impact fore of flowing layer against dead zone, *F*_d_ is the impact for of flowing layer against flexible barrier directly, *F*_p_ is the static pressure on flexible barrier. For slope pileup model, the pileup height keeps constant and reaches to maximum value *h*_cri_ at the end of the impact process. The static force of dead zone on flexible barrier is the main force. It indicates the impact force of granular flow with flow height *h*_0_ and flow velocity *v*_0_ reaches to the maximum value. The pileup types corresponding to different slope angle are shown in Fig 18. It is easy to be slope pileup model when the slope angle is small the angle of repose. It helps explain why *k* and *C*_d_ decrease in the increase of slope angle.

**Table 7:** Indexes of the impact process and pileup.

Slope angle/°	*h*_deposit_/m	*k*	*C*_d_
30	0.15	2.07	2.00
35	0.24	1.32	1.05
40	0.35	0.79	0.72
45	0.40	0.75	0.65
50	0.40	0.91	0.72

Since the dynamic tensile strength of the metal tow is about 1.1 to 1.2 times the static tensile strength [27], it is not necessary to increase the tensile strength of the support rope for the granular flow of uniform particle gradation, similar initial mass and potential energy, and the flow depth is more than twice the average particle size. It is relatively difficult to control gradation only in physical experiments rather than the bulk densities and the angles of repose. In contrast, the numerical analysis methods could control the initial accumulation density and particle size composition effectively. Further, particle size could not only have influence on the impact load of the granular flow through the interparticle collision and friction; it can also change the impact load of the granular flow due to the particle passing ratio. To study the comprehensive influences of the particle size distribution and mesh size on the interaction between the flexible barrier and granular flow, five different sizes of meshes were designed. The flexible mesh is hexagonal. The relationship between the mesh side length *l*_*m*_ and the mesh diameter *L*_*m*_ is:

$$L_{m}=2l_{m}\sin\;60{}^{\circ}.$$ (8)

**Fig 19:**
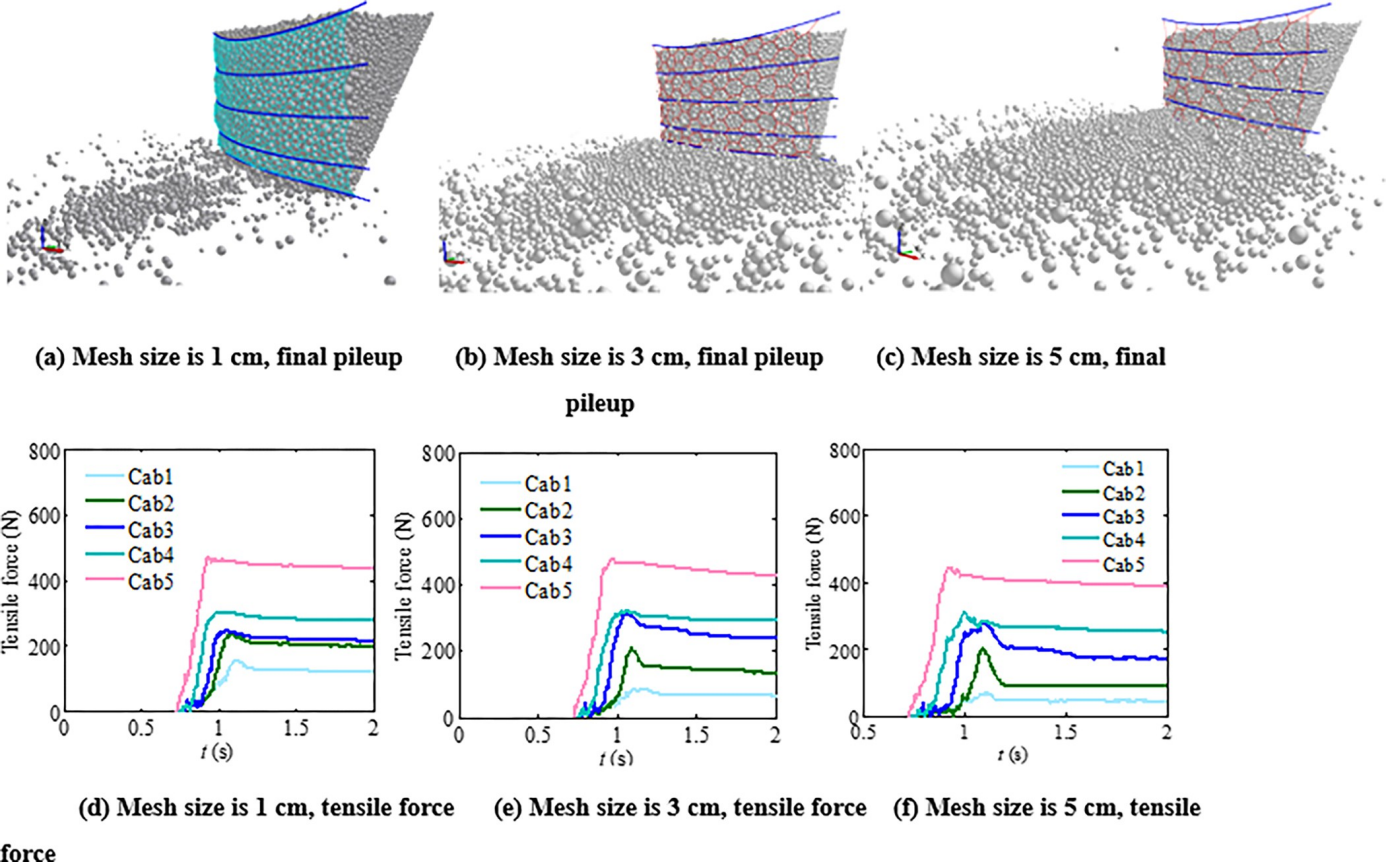
Final pileup of test materials and tensile force of the cables.

It could be obtained from the above formula that the ratio of mesh diameter to average particle size was close to 1~5 when the mesh side length was 1~5 cm. The final pileup of the granular flow and time interval curves of the cables’ tension force are shown in Fig 19. When the mesh diameter increases from one to five of the average particle diameters, the particle passing ratio increases from 5% to 33%. However, the maximum tensile force of the bottom cables remains 470~480 N. According to the physical experiments with different particle size ranges, the tensile force of the cables does not decrease with the increase of the ratio of mesh size to particle size under the following conditions: (1) the particle size distribution is uniform; (2) the initial mass of particle granular flow is similar, the flow depth is more than two times the average particle size; and (3) the ratio of flexible aperture to particle size is below 5.

### 3.2 Influences of particle segregation

**Fig 20:**
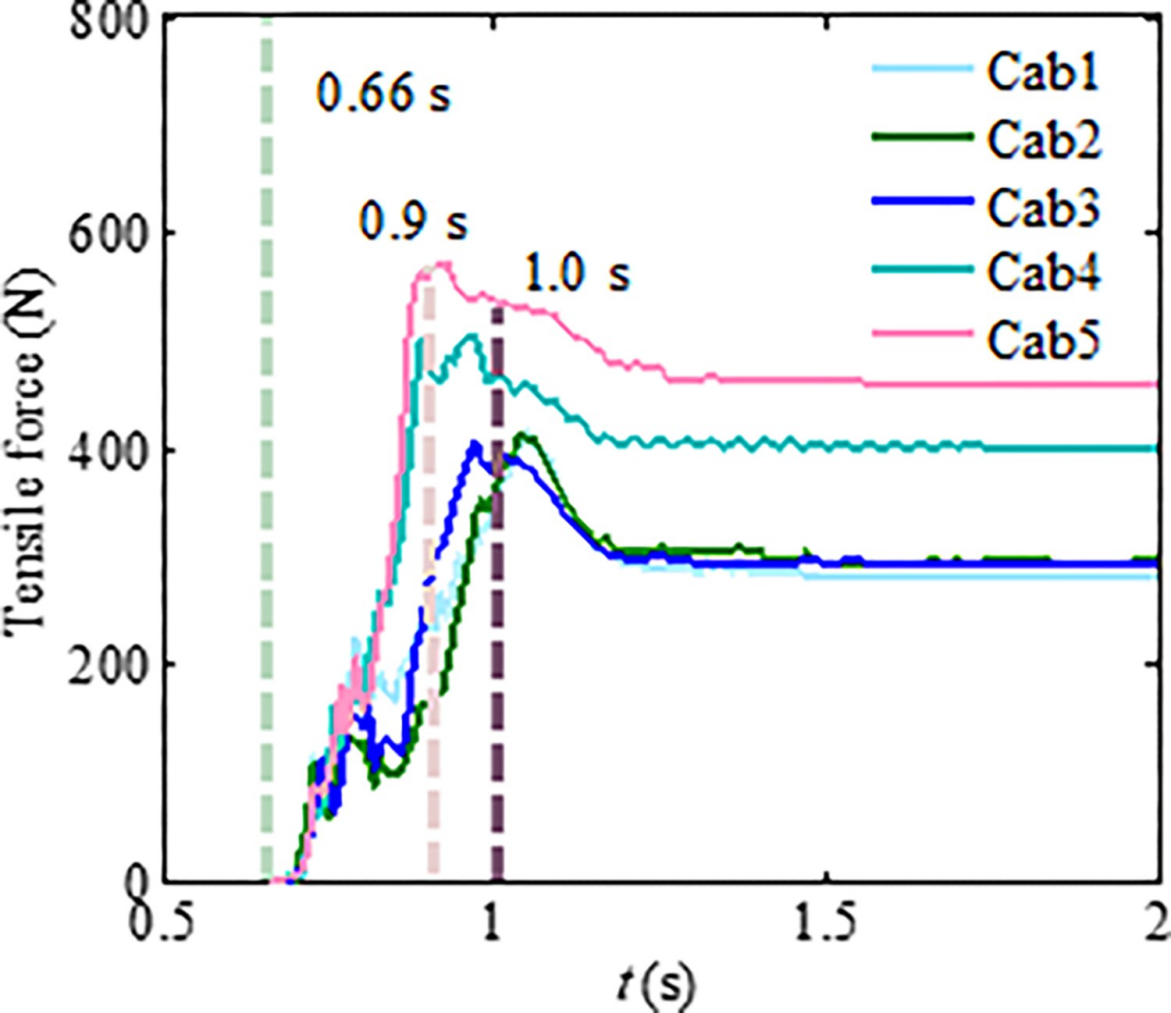
Tensile force of the cables.

**Fig 21:**
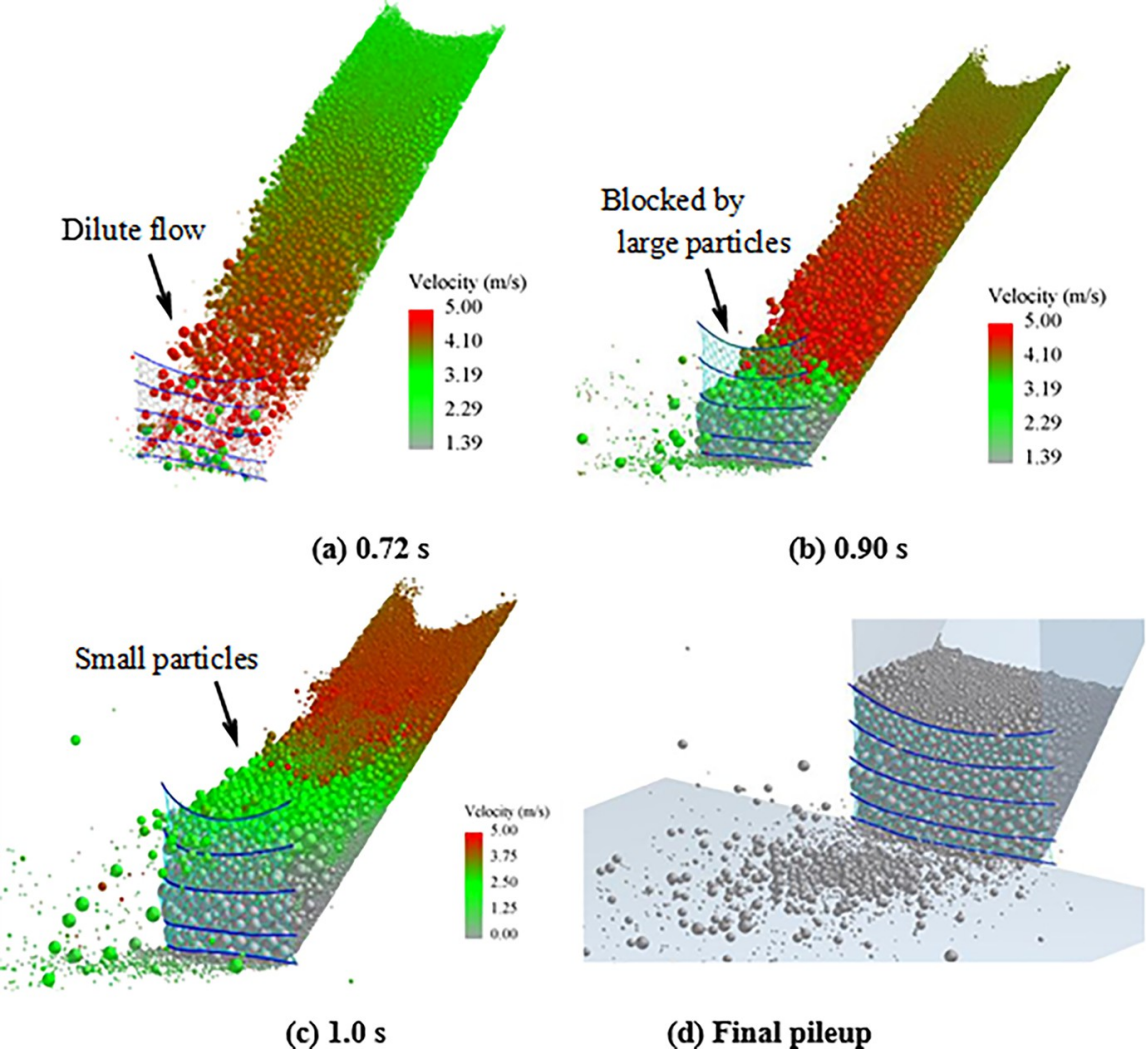
Processes of size segregation and impact of the granular flow.

Numerical analysis shows that particle segregation has influences on the flow regime of the granular flow, resulting in the impact load changes of the granular flow [26–28]. The main reason is that the large particles gradually appear at the leading edge of the granular flow because of the particle separation. This phenomenon enhances the fluidity of the granular flow. To investigate the influences of the particle separation on the tensile force of the flexible barrier, the test materials with a mix particle size are designed. The three types of particle size ranges are 0.5~2, 2~3, and 3~4 cm, respectively. Each type accounts for one-third of the total mass. The time interval curves of the cables’ tensile force are shown in Fig 20, which also shows that the particle separation effect leads to the impact load increase of the granular flow on the flexible barrier, as compared in Fig 13, without obvious particle segregation. The velocity distribution and final pileup of the granular flow during the impact on the flexible barrier are shown in Fig 21, which also shows that the large particles are mainly located in the leading edge part first because of the particle segregation. The main movement of these large particles is bouncing collision, as shown in Fig 21(A). Then, the large particles clog the mesh, as shown in Fig 21(B). The subsequent small particles cannot pass through the mesh, as shown in Fig 21(C) and 21(D). The maximum tensile force of the cables finally increases to 571 N, which is about 20% higher than that of the group without obvious particle segregation. Therefore, the tension of the support cables needs to be strengthened when there is an obvious segregation phenomenon in the flow process.

**Fig 22:**
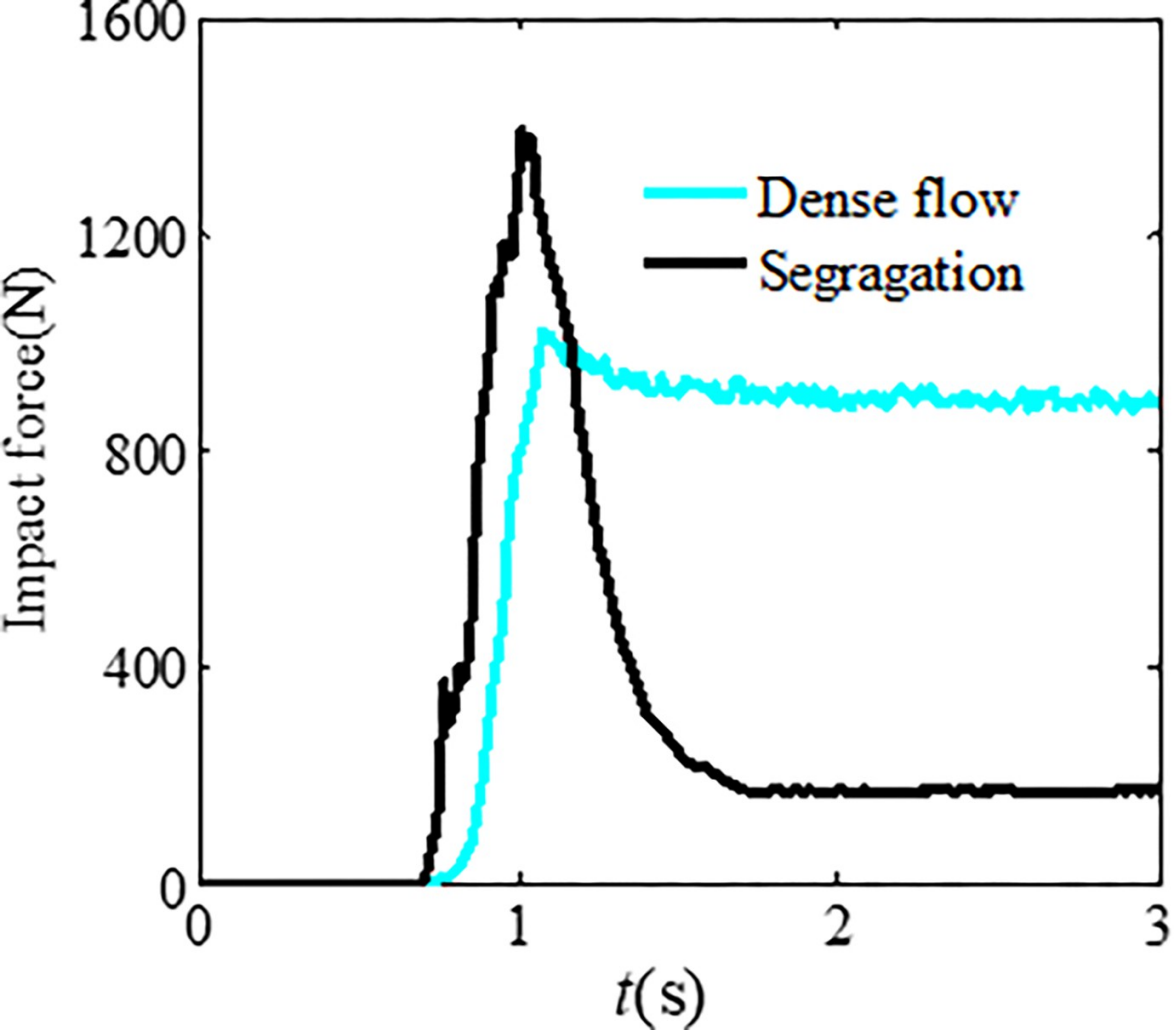
Differences in the impact force.

**Fig 23:**
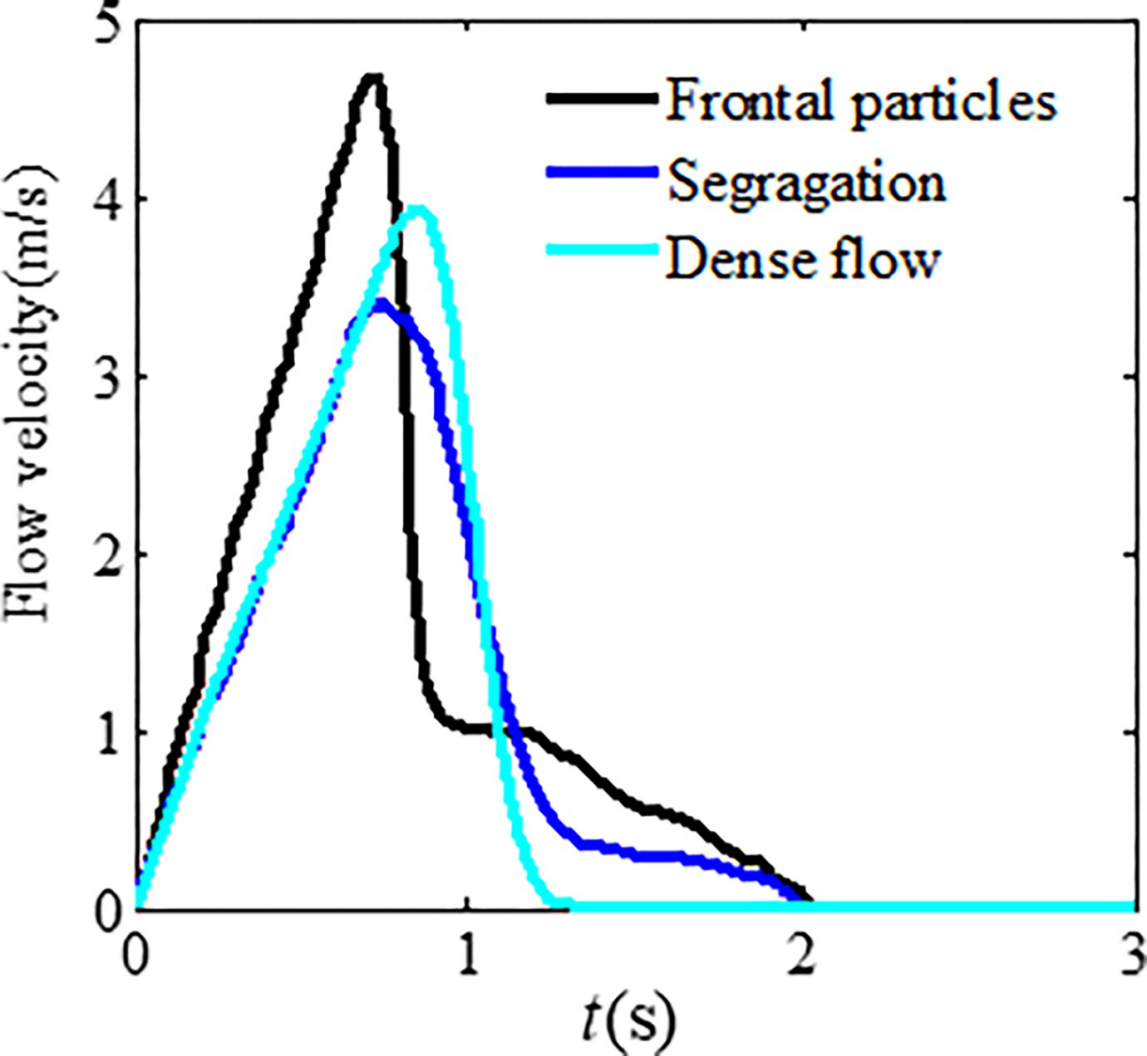
Differences in the average flow velocity.

Figs 22 and 23 show the differences in impact force and flow velocity. Fig 22 indicates that granular flow with particle segregation has a greater impact force than dense flow, and the impact time occurs earlier than that of dense flow. According to Fig 23, the average flow velocity of the leading particles is greater than that of dense flow, indicating that larger particles at the front have greater velocity. However, the average flow velocity of dense flow is greater than that of segregated flow. This could be due to smaller particles in segregated flow having a smaller velocity.

## 4 Influences of the dilute flow regime on the interaction between flexible barrier and granular flow

**Fig 24:**
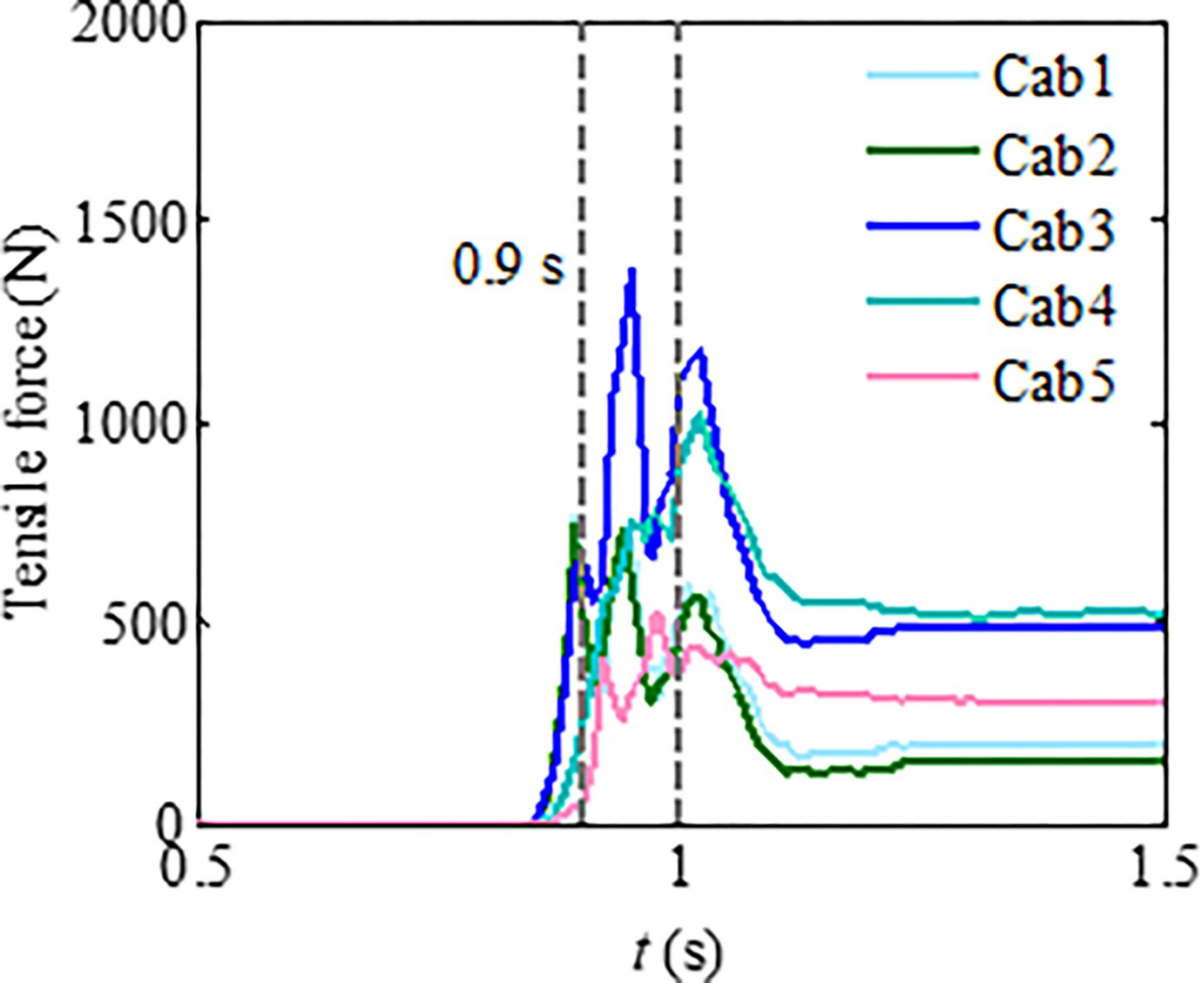
Tensile force of the cables as the mesh size is 3 cm.

**Fig 25:**
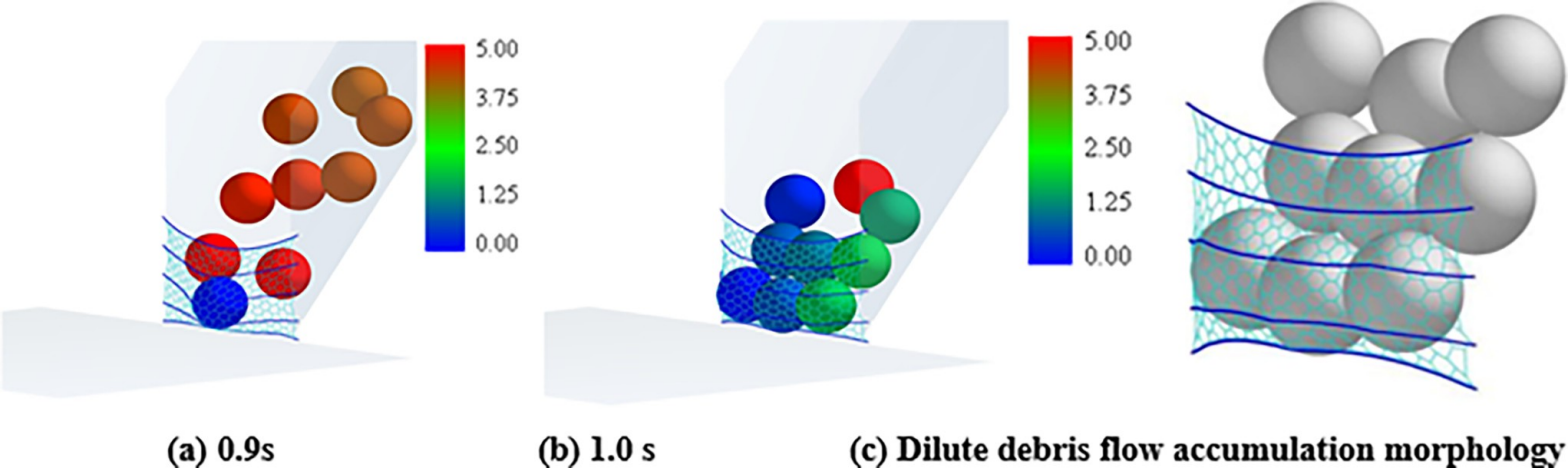
Processes of impact of the granular flow (unit: m/s).

The granular flow presents an obvious dilute flow characteristic by increasing particle size. A total of nine particles were generated to impact the flexible barrier based on the same total mass of previous tests. In addition, in order to study the differences between the impact of dilute flow and rockfall on a flexible barrier, a group of rockfall impact flexible barriers with a total mass of 74.4 kg was designed. The impact time interval curves and process of the dilute granular flow are shown in Figs 24 and 25, respectively. The motion of the dilute flow is mainly the collision and jump instead of the friction. After the particles impact the flexible barrier, the impact position is between Cab3-Cab4. Therefore, the maximum impact load of dilute granular flow is located in the middle of the flexible barrier instead of the bottom cable. The flexible barrier was subjected to obvious impulse loads, and the maximum tensile force is about three times that of corresponding to the dense flow impacting the flexible barrier.

**Fig 26:**
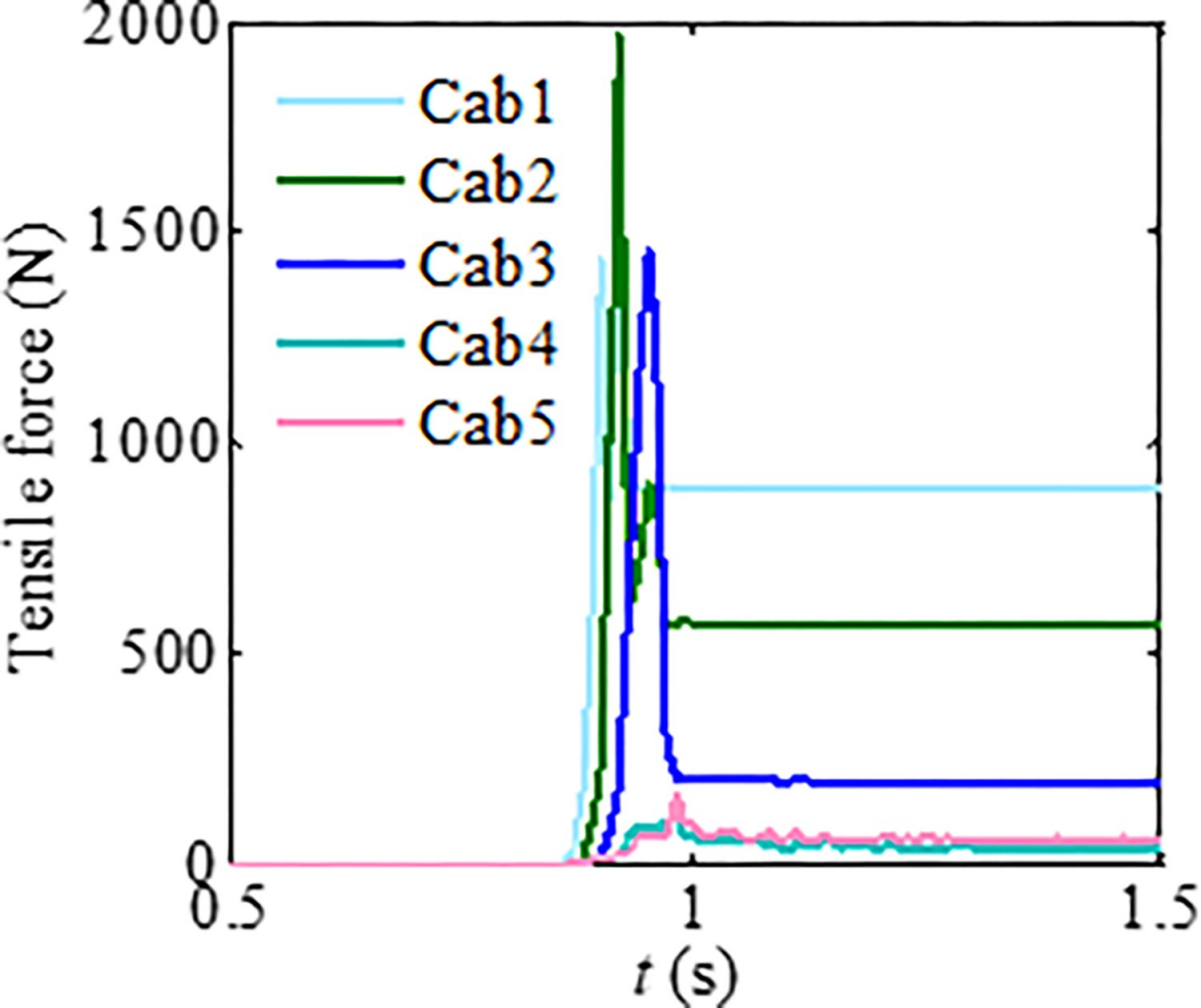
Tensile of the cables when the granular flow impacts the barrier.

**Fig 27:**
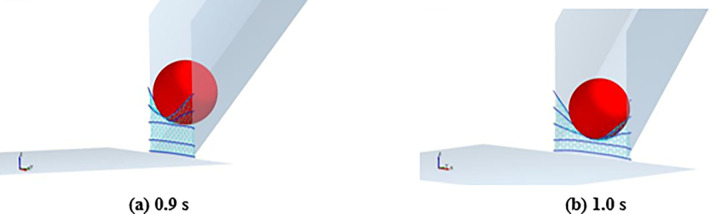
Processes of impact of granular flow.

The tension time interval curves of the cables and impact process of the rockfall are, respectively, presented in Figs 26 and 27. As shown in Fig 26, the cables have a more obvious impulse tension load. The impact load is located on the upper cables corresponding to the rockfall impact position, as shown in Fig 27(A). The top cables were broken, and the flexible mesh fails under the impulse impact load, as shown in Fig 27(B). Comparing the time interval curves of the cables, as shown in Fig 24, the design of the flexible barrier impacted by the dilute flow needs to adopt the design method of the flexible barrier impacted by a rockfall [29].

## 5 Preliminary suggestions for the design of flexible barrier impacted by a granular flow

**Fig 28:**
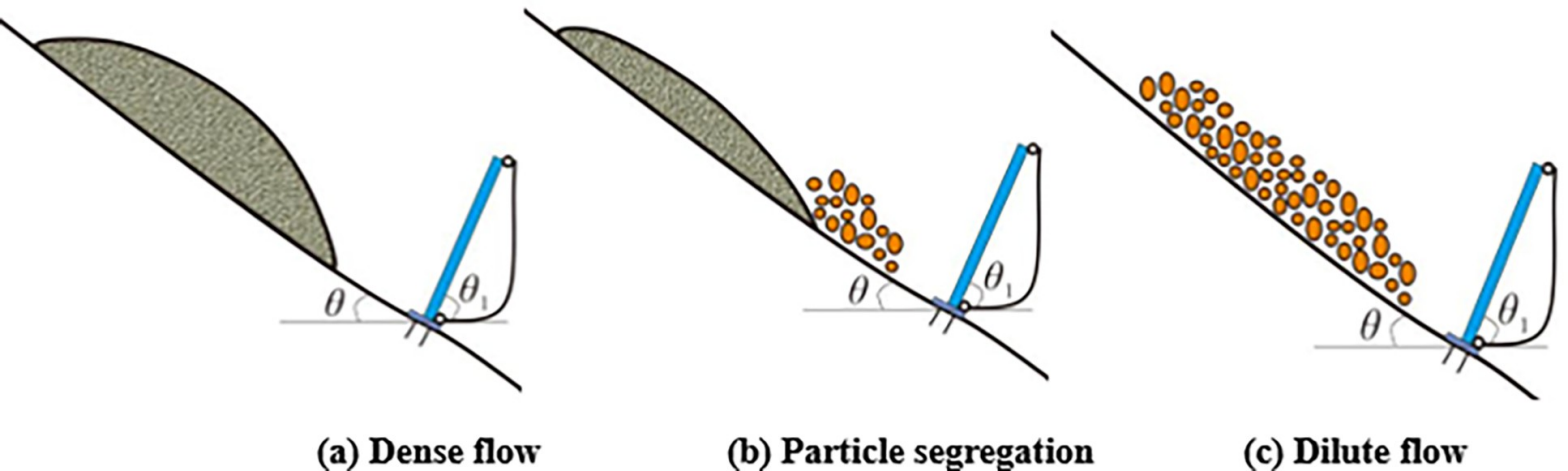
Impact of the granular flows with different flow regimes.

It could be found that the dense flow particle movement is mainly dominated by friction and collision by comparing the interactions of flexible barriers between a dense flow and dilute flow. The impact of the dense flow on a flexible barrier is plotted in Fig 28(A). In this case, the maximum tensile force generally occurred at the bottom cables of a flexible barrier under the impact of a dense flow.

The dynamic indexes of the impact process corresponding to granular flows with different flow regimes are listed in Table 8. If there is obvious particle segregation during the granular flow movement, the large particles at the leading edge will gradually impact the flexible barrier, as shown in Fig 28(B). It leads to an increase in the maximum tensile force of the cables, as shown in Table 8 and Fig 20, which also shows that the flexible barrier positions, which need to increase tensile strength under the impact of a dense flow and a dilute flow, are also inconsistent. For dense flow protection, the bottom of the support rope should be strengthened. It is necessary to focus on enhancing the tensile capacity of the middle and top cables of the flexible barrier to intercept a wide gradation granular flow with an obvious particle segregation. To prevent the bullet effect of dilute flow, it is more suitable for designing the flexible barrier based on the design method of the flexible barrier to intercept a rockfall. The impact of a dilute flow on a flexible barrier is shown in Fig 28(C). In terms of the impact duration, the flow regimes could also lead to some differences. The impact duration of dense flow is longest; it roughly reaches to 1 s, as shown in Fig 13. Fig 26 shows that the shortest duration is the impact of rockfall, which is about 0.3s. The duration of dilute flow is between the above. These differences indicate that the impact load on a flexible barrier will increase because of the shorter impact duration based on the law of conservation of momentum.

**Table 8:** Dynamic indexes of the impact process.

Flow regime	Maximum impact force	Flow height/m	Flow velocity/m·s^-1^	Froude number	*k*	*C*_d_
Dense flow	697.2	0.052	4.97	8.68	0.91	0.72
Particle segregation	1389.8	0.048	5.34	7.93	1.8	1.35
Dilute flow	1643.7	/	5.67	/	/	/
Rockfall	2089.3	/	6.23	/	/	/

In summary, the influences of particle size on the impact load of a granular flow are as follows: The impact load of the collapse granular flow gradually increases in the changes in the flow states from dense flow to dilute flow. The maximum tensile force of the cable moves from the lower part of cables to the upper part of the cables without consideration to the mass and potential energy changes during the movement as well as the influences of the site conditions on the movement characteristics of the granular flow under the conditions of the same initial mass and potential energy. The differences of the design of the flexible barrier under the impact of the granular flow with different flow regimes are listed in Table 9.

**Table 9:** Design of the flexible barrier to intercept a granular flow.

Flow Regimes	Grain Gradation	Froude Number	Ratio of the Particle Size to Flow Height	Characteristics of Movement	Initial Impact Position	Design Method
Density flow	Uniform gradation	<2	>2	Friction and collision	Bottom	Hydro-dynamic model or Hydro-static model
Density flow-dilute flow	Wide gradation	2~10	/	Friction, jump, collision	Top/middle	Empirical coefficient method
Dilute flow	Uniform gradation/Wide gradation	>10	<2	Jump, collision	Top/middle	Impact energy matching

## 6 Conclusion

This study analyzed the influences of the changes in the flow regimes on the interaction characteristics of the granular flow impacting the flexible barrier combined with the physical model and discrete element simulation tests. The main conclusions are as follows:

1. The increase in ratio of mesh size to particle size will not lead to a significant increase in the tensile force of the flexible barrier based on the numerical and physical results under following conditions: a) the particle size distribution of the granular flow is uniform; b) the flow state is dense flow; the initial mass and potential energy are similar; c) the flow depth is more than twice the average particle size; d) the ratio of the mesh size to the particle size is below 5. In this case, the flow depth and flow velocity of the granular flow could be used for estimating the impact load of the granular flow against a flexible barrier.
2. According to the numerical results, it is necessary to enhance the tensile strength of the middle and upper cables when the particle size distribution of the granular flow is wide, and there is an obvious particle segregation in the flow process. When the flow state of granular flow is an obvious dilute flow, the flexible barrier structure should be designed according to the energy level standard of a rockfall prevention barrier.
3. In this study, the maximum tensile force of the cables impact by a wide gradation granular flow is about four times that of the maximum tensile force of the cables impact by a dense flow with a uniform gradation granular flow. How to estimate quantitatively the influences of the particle segregation on the interaction between the granular flow and the flexible barrier is worthy of further study.

Dry granular particle materials were used in this study. The impacting dynamic characteristic was affected by other factors such as the dynamic water pressure, the rheological characteristics of granular flow, and water content. Therefore, the influence of the corresponding flow states on the interaction with a flexible barrier still needs further research.
